# Catalytically inactive Dnmt3b rescues mouse embryonic development by accessory and repressive functions

**DOI:** 10.1038/s41467-019-12355-7

**Published:** 2019-09-26

**Authors:** Pawel Nowialis, Katarina Lopusna, Jana Opavska, Staci L. Haney, Ajay Abraham, Peike Sheng, Alberto Riva, Amarnath Natarajan, Olga Guryanova, Melanie Simpson, Ryan Hlady, Mingyi Xie, Rene Opavsky

**Affiliations:** 1 Department of Anatomy and Cell Biology, University of Florida College of Medicine, 2033 Mowry Rd, CGRC 258, Gainesville, FL 32610 USA; 2 UF Health Cancer Center, University of Florida, Gainesville, FL 32610 USA; 3 Department of Internal Medicine, University of Nebraska Medical Center, 985950 Nebraska Medical Center, Omaha, NE 68198 USA; 4 Department of Biochemistry and Molecular Biology, University of Florida College of Medicine, 2033 Mowry Rd, CGRC 263, Gainesville, FL 32610 USA; 5 ICBR Bioinformatics, Cancer and Genetics Research Complex, University of Florida, P.O. Box 103622, Gainesville, FL 32610 USA; 6 University of Nebraska Medical Center, The Eppley Institute for Research in Cancer and Allied Diseases, Fred & Pamela Buffett Cancer Center, 986805 Nebraska Medical Center, Omaha, NE 68198 USA; 7 Department of Pharmacology and Therapeutics, University of Florida College of Medicine, 2033 Mowry Rd, CGRC 260, Gainesville, FL 32610 USA; 8 Molecular and Structural Biochemistry, NC State University, Raleigh, NC 27695 USA; 9 Department of Molecular Pharmacology and Experimental Therapeutics, Mayo Clinic, Rochester, MN 55901 USA

**Keywords:** Embryogenesis, Epigenetics, Gene expression, DNA methylation, Transcription

## Abstract

DNA methylation regulates gene expression in a variety of processes, including mouse embryonic development. Four catalytically active enzymes function in mice as DNA methyltransferases (Dnmts) and as transcriptional regulators. Inactivation of Dnmt3b results in mouse embryonic lethality, but which activities are involved is unclear. Here we show that catalytically inactive Dnmt3b restores a majority of methylation and expression changes deregulated in the absence of Dnmt3b, and as a result, mice survive embryonic development. Thus, Dnmt3b functions as an accessory cofactor supporting catalytic activities performed by other Dnmts. We further demonstrate that Dnmt3b is linked to a control of major developmental pathways, including Wnt and hedgehog signaling. Dnmt3b directly represses Wnt9b whose aberrant up-regulation contributes to embryonic lethality of Dnmt3b knockout embryos. Our results highlight that Dnmt3b is a multifaceted protein that serves as an enzyme, an accessory factor for other methyltransferases, and as a transcriptional repressor in mouse embryogenesis.

## Abstract (web summary)

The family of DNA methyltransferases (Dnmts) consists of four catalytically active enzymes that catalyze DNA methylation and also play a role as transcriptional regulators. Here the authors show that catalytically inactive Dnmt3b rescues a majority of methylation and expression changes in the absence of Dnmt3b during mouse embryonic development.

## Introduction

DNA methylation of CpG dinucleotides is a covalent modification involved in regulation of gene expression in mammalian cells. It has suppressive effects on gene transcription, especially in CpG islands (CGIs) of gene promoters or in enhancers where it is often associated with repressive histone modifications such as H3K9me3 and H3K27me3 (refs. ^1,2^). Methylation can also enhance transcription by promoting more efficient binding of transcription factors to their recognition sites ^3,4^ and by restricting the activation of alternative promoters within gene bodies^4^. Thus, effects of DNA methylation on gene expression are complex and context-dependent.

The family of DNA methyltransferases (Dnmts) consists of four catalytically active enzymes (Dnmt1, Dnmt3a, Dnmt3b, and Dnmt3c) and one catalytically inactive cofactor (Dnmt3L) in mice. All Dnmts participate in generation and maintenance of methylation highlighting complexity of this epigenetic phenomenon^5–7^. Dnmt3L is an accessory protein that lacks catalytic activity (CA) but is critical for induction of de novo methylation by linking Dnmt3a and Dnmt3b to chromatin through unmethylated H3 lysine 4 (ref. ^8^). Dnmt3c suppresses transposon activity specifically in male germ cells^9^. Apart from CA, Dnmts suppress transcription through repression by association with HDACs, MBDs, Rb, and other Dnmts^10^. In addition, Dnmts can associate with areas of active gene transcription. DNMT3B selectively binds to the bodies of actively transcribed genes through the interaction of its PWWP domain with histone H3 trimethylated at lysine 36, and plays a role in their preferential methylation in embryonic stem cells^11^.

DNA methylation plays a major role in development from conception. After fertilization, methylation is erased at the blastocyst stage, and then re-established between 4.5 and 6.5 days post coitum in murine epiblast cells by the combined action of DNMT3A and DNMT3B. Newly generated methylation patterns form a basis for tissue-specific expression, and are utilized in cell differentiation and organogenesis later during embryogenesis^12,13^. Consistently with the importance of methylation, inactivation of Dnmt1 or Dnmt3b is embryonically lethal at E9.5 and ~E11.5–15.5, respectively^5,6,14^. *Dnmt3a*^*−/−*^ mice die shortly after the birth^5^. The contribution of Dnmt3b to genome methylation is greater than that of Dnmt3a and consists of specific methylation of sequences associated with developmental and imprinted genes, and X chromosome inactivation^13^.

Because of numerous activities including catalytic and repressive functions, as well as expression of numerous DNMT3b isoforms, it is difficult to understand which physiological processes solely depend on Dnmt3b’s CA^15,16^. To understand contribution of Dnmt3b’s CA activity to embryogenesis, we generated and analyzed mice expressing catalytically inactive Dnmt3b allele (*Dnmt3b*^*CI*^) from the endogenous locus. *Dnmt3b*^*CI/CI*^ survive embryonic development because catalytically inactive Dnmt3b restores a majority of molecular changes observed in *Dnmt3b*^*−/−*^ mice. We further show that aberrant activation of Wnt signaling contributes to lethality of *Dnmt3b*^*−/−*^ embryos and that Dnmt3b inhibits this pathway by repressing Wnt9b. Thus, Dnmt3b functions as an accessory factor to other Dnmts and as a transcriptional repressor in mouse embryogenesis.

## Results

### Catalytic activity of Dnmt3b is dispensable for embryogenesis

**Fig. 1:**
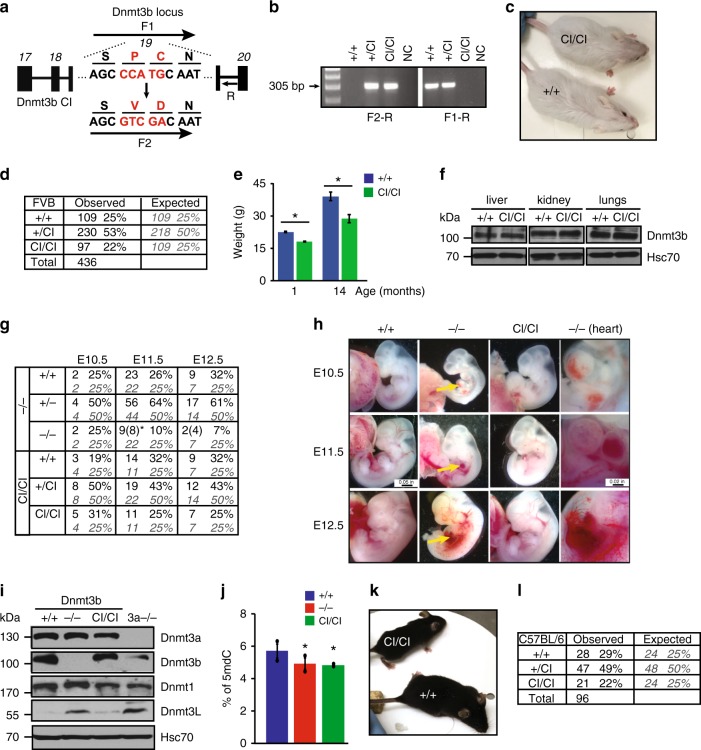
Catalytically inactive Dnmt3b is sufficient for normal mouse embryogenesis. **a** The schematic illustrates exons 17–20 (black boxes) of the mouse *Dnmt3b*, a portion with nucleotide and amino acid sequences in exon 19 encoding wild type (top) and sequence encoding amino acid substitution: PC to VD inactivating catalytic activity that was used to generate *Dnmt3b*^*CI*^ allele (bottom). Position of genotyping primers is indicated by arrows. F1-R detects wild-type sequence; F2-R detects mutated sequence. **b** PCR genotyping assay performed on DNA isolated from *Dnmt3b*^*+/+*^ (+/+), *Dnmt3b*^*+/CI*^ (+/CI) and *Dnmt3b*^*CI/CI*^ (CI/CI) mice. F1-R and F2-R represents primer pairs detecting wild type and CI allele, respectively. NC, no template control. **c** Micrograph of 6-week-old +/+ and CI/CI mice. **d** Genotypic analysis of offspring derived from *Dnmt3b*^*+/CI*^ intercrosses (FVB strain). **e** The weight of *Dnmt3b*^*+/+*^ (+/+) and *Dnmt3b*^*CI/CI*^ (CI/CI) mice measured at 1 (*n* = 16) and 14 months (*n* = 4). Data are presented as mean ± SEM, *p < 0.001 (two-tailed Student’s *t*-test). **f** Immunoblot showing Dnmt3b protein levels in liver, kidney, and lungs of 8 weeks old +/+ and CI/CI mice. Hsc70 served as a loading control. **g** Genotypic analysis of embryos isolated from intercrosses of *Dnmt3b*^*+/−*^ and *Dnmt3b*^*+/CI*^ mice at E10.5, E11.5, and E12.5. Bold font indicates observed numbers and gray italic indicates expected number. Numbers in brackets denote dead embryos. *p < 0.01 (*χ*^2^-test). **h** Gross morphology of *Dnmt3b*^*+/+*^, *Dnmt3b*^*−/−*^ and *Dnmt3b*^*CI/CI*^ E10.5, E11.5, and E12.5 embryos (magnification ×8). Arrows point to hemorrhages and pericard edema of *Dnmt3b*^*−/−*^ mice. Higher-magnification (×40) images of hearts are shown in the right panel. **i** Immunoblot showing Dnmt3a, Dnmt3b, Dnmt1, Dnmt3L, and Hsc70 (loading control) protein levels in E11.5 *Dnmt3b*^*+/+*^ (+/+), *Dnmt3b*^*−/−*^ (^*−/−*^), *Dnmt3b*^*CI/CI*^ (CI/CI), and *Dnmt3a*^*−/−*^ (3a^*−/−*^) whole embryo lysates. This labeling of genetic groups is used in all subsequent figures unless noted otherwise. **j** Percentage of methylated cytosines in genomic DNA in *Dnmt3b*^*+/+*^ (+/+), *Dnmt3b*^*−/−*^ (^*−/−*^), *Dnmt3b*^*CI/CI*^ (CI/CI) E11.5 embryos (*n* = 2) as determined by mass spectrometry. Data are presented as mean ± SEM. *p < 0.05 (two-tailed Student’s *t*-test). **k** Micrograph of 6-week-old +/+ and CI/CI mice (C57BL/6 strain). **l** Genotypic analysis of offspring derived from intercrosses of *Dnmt3b*^*+/CI*^ mice (C57BL/6 background). Source data are provided as a Source Data file

To address whether Dnmt3b’s CA is important for mouse embryogenesis, we sought to generate Dnmt3b allele lacking CA. A replacement of cysteine at position 657 by a serine or threonine in mouse Dnmt3b coding sequence (CDS) has been shown to abolish CA without compromising other functions^17,18^. Similarly, double amino acid (aa) substitution P705V/C706D in the CDS of Dnmt3a inactivates its CA^19^. Therefore, we considered substitution Dnmt3b^P656,C657^ (catalytic site corresponding to Dnmt3a^P705,C706^) for valine and aspartate, respectively. Such substitution is predicted to disable first two steps (nucleophilic addition and methyl transfer) of the three-step process required for CA, thus rendering Dnmt3b^P656V/C657D^ protein catalytically inactive (CI; referred to here as Dnmt3b^CI^) (Fig. 1a, Supplementary Fig. 1).

To test CA, we expressed both *wild-type* (Dnmt3b^WT^) and Dnmt3b^CI^ in *Dnmt3a*^*−/−*^;*Dnmt3b*^*−/−*^ mouse lymphoma cell line^20^ and analyzed global methylation by reduced representation bisulfite sequencing (RRBS). In this assay, the ability of Dnmt3b^CI^ to induce differentially methylated regions (DMRs; defined as ≥30% methylation change in three consecutive cytosines in ≤100 bp) was severely diminished (~10-fold) relative to Dnmt3b^WT^ (Supplementary Fig. 2). Residual activity of Dnmt3b^CI^ may stem from complexing with intracellular Dnmts, such as Dnmt1. Although we cannot rule out that Dnmt3b^CI^ retains some residual CA activity, our bioinformatics and functional assays strongly suggest that this protein lacks CA.

To generate mice expressing Dnmt3b^CI^ protein from the endogenous locus, we utilized CRISPR/Cas9 homology-directed repair to target exon 19 of Dnmt3b by co-injection of mRNA, sgRNA, and donor oligo into FVB mouse zygotes (Fig. 1a). A proper targeting was confirmed by PCR-based genotyping followed by sequencing. *Dnmt3b*^*+/CI*^ founders were born without any detectable phenotypes with long lifespan. Subsequent analysis showed no phenotypic differences; therefore, we further do not distinguish between founder-derived offspring.

Interbreeding of *Dnmt3b*^*+/CI*^ mice yielded *Dnmt3b*^*CI/CI*^ mice that were surprisingly alive at weaning age although the genetic ratios were slightly lower than expected (Fig. 1b–d). Interestingly, *Dnmt3b*^*CI/CI*^ mice were ~20% smaller than their *Dnmt3b*^*+/+*^ littermates at weaning and such differences persisted throughout their lives (Fig. 1e). Both male and female *Dnmt3b*^*CI/CI*^ mice are fertile and have normal lifespan.

Dnmt3b^CI^ levels were indistinguishable from Dnmt3b^WT^ levels in adult tissues suggesting that an introduction of aa substitutions did not adversely affect regulation of Dnmt3b expression or protein stability (Fig. 1f).

Because embryonic lethality of *Dnmt3b*^*−/−*^ mice at E11.5–15.5 was previously addressed using unrelated mouse strains^5,6^, we next asked whether *Dnmt3b*^*−/−*^ embryos derived from the FVB genetic background can, like *Dnmt3b*^*CI/CI*^ mice, survive embryogenesis. We found that *Dnmt3b*^*−/−*^ embryos are underrepresented at E11.5 and E12.5, suggesting embryonic lethality in this mouse strain (Fig. 1g). Morphological analysis revealed heart defects, including enlarged pericard with edema, and hemorrhage in *Dnmt3b*^*−/−*^ but not in *Dnmt3b*^*CI/CI*^ embryos (Fig. 1h, Supplementary Fig. 3).

Like in adult tissues, Dnmt3b^CI^ protein levels in *Dnmt3b*^*CI/CI*^ embryos were comparable to WT counterparts. Dnmt1 and Dnmt3a were also unchanged, suggesting that the observed rescue of embryogenesis is not due to compensation by these proteins (Fig. 1i). Interestingly, Dnmt3L was up-regulated in *Dnmt3b*^*−/−*^ but not in *Dnmt3b*^*CI/CI*^ embryos suggesting potential functional compensation for the loss of Dnmt3b (Fig. 1i). Global level of 5-methylcytosine in *Dnmt3b*^*CI/CI*^ was reduced relative to *Dnmt3b*^*+/+*^ but not to *Dnmt3b*^*−/−*^ E11.5 embryos (Fig. 1j). Furthermore, methylation of two Dnmt3b target genes, *Ddx4* (ref. ^13^) and *Ment*^21^, was similarly reduced in both *Dnmt3b*^*−/−*^ and *Dnmt3b*^*CI/CI*^ E11.5 embryos (Supplementary Figs. 4 and 5). These data further suggest that Dnmt3b^CI^ lacks CA in vivo.

To determine if the rescue of embryogenesis is limited to the FVB strain, we generated independent mouse lines through targeting of C57BL/6 strain zygotes as outlined in Fig. 1a. Interbreeding of *Dnmt3b*^*+/CI*^ mice yielded viable *Dnmt3b*^*CI/CI*^ pups (Fig. 1k, l), demonstrating that the rescue of embryogenesis by Dnmt3b^CI^ is not strain-specific.

Altogether, these results demonstrate that CA of Dnmt3b is, surprisingly, dispensable for mouse embryogenesis.

### Accessory activity of Dnmt3b restores global methylation

**Fig. 2:**
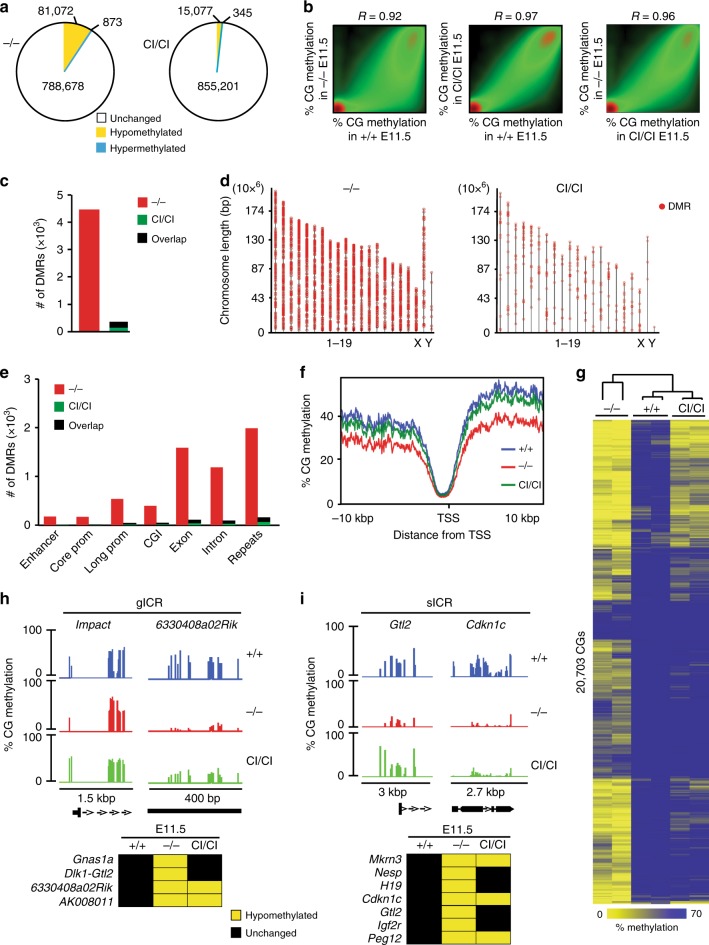
Dnmt3b^CI^ restores deregulated methylation in *Dnmt3b*^*−/−*^ embryos. **a** Total number of differentially methylated CGs in *Dnmt3b*^*−/−*^ and *Dnmt3b*^*CI/CI*^ E11.5 embryos relative to *Dnmt3b*^*+/+*^ counterparts as analyzed by RRBS (*n* = 2 each). Differential CG methylation is defined as ≥30% change. **b** Pairwise comparison of CG methylation between *Dnmt3b*^*+/+*^, *Dnmt3b*^*−/−*^, and *Dnmt3b*^*CI/CI*^ E11.5 embryos. The density of points increases from green to red. *R* values represent Pearson correlation coefficients. **c** The number of DMRs (≥3 consecutive DMCs in the same direction in the distance ≤200 bp, average DMC methylation change ≥30%, *p* < 0.05) identified in *Dnmt3b*^*−/−*^ and *Dnmt3b*^*CI/CI*^ E11.5 embryos relative to *Dnmt3b*^*+/+*^ counterparts. Overlapping DMRs between *Dnmt3b*^*−/−*^ and *Dnmt3b*^*CI/CI*^ are marked with black. **d** Distribution of DMRs on chromosomes identified in *Dnmt3b*^*−/−*^ and *Dnmt3b*^*CI/CI*^ E11.5 embryos relative to *Dnmt3b*^*+/+*^ counterparts. Only regions with sufficient coverage are shown. **e** The number of DMRs associated with enhancers, core promoters (−300 to +150 bp relative to TSS), long promoters (−1500 to +500 bp), CGIs, exons, introns, and repeats identified in *Dnmt3b*^*−/−*^ and *Dnmt3b*^*CI/CI*^ E11.5 embryos relative to *Dnmt3b*^*+/+*^ counterparts. **f** Distribution of CG methylation in the region from −10 to +10 kbp around TSS in *Dnmt3b*^*+/+*^, *Dnmt3b*^*−/−*^, and *Dnmt3b*^*CI/CI*^ E11.5 embryos. **g** Hierarchical clustering of CG methylation in E11.5 embryos of indicated genotypes. Heat map shows 20,703 CGs included in all DMRs identified in *Dnmt3b*^*−/−*^ *and Dnmt3b*^*CI/CI*^ embryos. **h** Percentage of methylation at individual CGs in germline Imprinted Control Region (gICR) *Impact* (chr18: 12,973,600–12,972,150) and *6330408a02Rik* (chr7: 13,261,170–13,261,570) as visualized by IGB software (top). Summary of all hypomethylated gICRs identified in *Dnmt3b*^*−/−*^ and *Dnmt3b*^*CI/CI*^ embryos (bottom). **i** Percentage of methylation at individual CGs in somatic Imprinted Control Region (sICR) Gtl2 (chr12: 109,539,000–109,542,000) and Cdkn1c (chr7:143,459,000–143,461,700) as visualized by IGB software (top). Summary of all hypomethylated sICRs identified in *Dnmt3b*^*−/−*^ and *Dnmt3b*^*CI/CI*^ embryos (bottom). Source data are provided as a Source Data file

To address the effects of Dnmt3b deficiency, we next analyzed global DNA methylation patterns in *Dnmt3b*^*+/+*^, *Dnmt3b*^*−/−*^, and *Dnmt3b*^*CI/CI*^ embryos at E11.5. RRBS yielded methylation information on more than million CGs with coverage ≥15×, out of which >8 × 10^5^ CGs present in all samples were used for the further analysis (Supplementary Fig. 6). Most of the differentially methylated cytosines (DMCs) in *Dnmt3b*^*−/−*^ embryos were hypomethylated relative to WT counterparts, but these changes were substantially reduced in *Dnmt3b*^*CI/CI*^ embryos (Fig. 2a, b). Further analysis revealed >4000 hypomethylated and no hypermethylated DMRs in *Dnmt3b*^*−/−*^ embryos (Fig. 2c). In contrast, only 315 hypomethylated DMRs were identified in E11.5 *Dnmt3b*^*CI/CI*^ embryos, most of which overlapped with DMRs found in *Dnmt3b*^*−/−*^ embryos (Fig. 2c). Thus, the presence of Dnmt3b^CI^ resulted in restoration of DNA methylation of ~95% (4133/4338) of DMRs that was lost in *Dnmt3b*^*−/−*^ embryos. Such effect was observed across the genome and as a result methylation of *Dnmt3b*^*CI/CI*^ embryos clusters apart from *Dnmt3b*^*−/−*^ and close to WT (Fig. 2d–g, Supplementary Fig. 7, Supplementary Data 1).

Dnmt3b is dispensable for methylation of germline imprinted control regions (gICRs) but involved in methylation of secondary somatic ICRs (sICRs)^13,22–24^. To gain further insight into Dnmt3b functions, we next analyzed methylation of 22 gICRs and 9 sICRs^22–24^. As expected, Dnmt3b was dispensable for methylation of majority of gICRs including *Impact* (Fig. 2h, Supplementary Data 2). However, we observed substantial hypomethylation in *Gnas1a, Dlk1-Gtl2, 6330408a02Rik*, and *AK008011* gICRs in E11.5 *Dnmt3b*^*−/−*^ embryos. Methylation was restored to WT levels only for *Gnas1a* and *Dlk1-Gtl2* in *Dnmt3b*^*CI/CI*^ embryos, suggesting that CA of Dnmt3b is critical for *6330408a02Rik* and *AK008011* loci (Fig. 2h, Supplementary Fig. 8, Supplementary Data 2).

Similarly, we also observed hypomethylation of 7/9 sICRs (*Mkrn3*, *Nesp, Cdkn1c, Gtl2, H19, Peg12, Igf2r*) in *Dnmt3b*^*−/−*^ embryos that was restored only for *H19, Nesp*, *Gtl2,* and *Igf2r* in *Dnmt3b*^*CI/CI*^ embryo (Fig. 2i, Supplementary Fig. 8). The remaining sICRs (diffuse DMRs- *Gtl2-Mirg* and *Eif2c2)* were not targeted by Dnmt3b, since they were not changed in methylation (Supplementary Data 2).

Altogether, our analysis revealed that a vast majority of methylation changes observed in *Dnmt3b*^*−/−*^ embryos is restored in *Dnmt3b*^*CI/CI*^ to WT levels consistently with the successful rescue of embryogenesis. Thus, Dnmt3b plays a role as accessory factor for DNA methylation independent of its intrinsic catalytic activity.

### Activation of demethylated promoters lacking histone marks

**Fig. 3:**
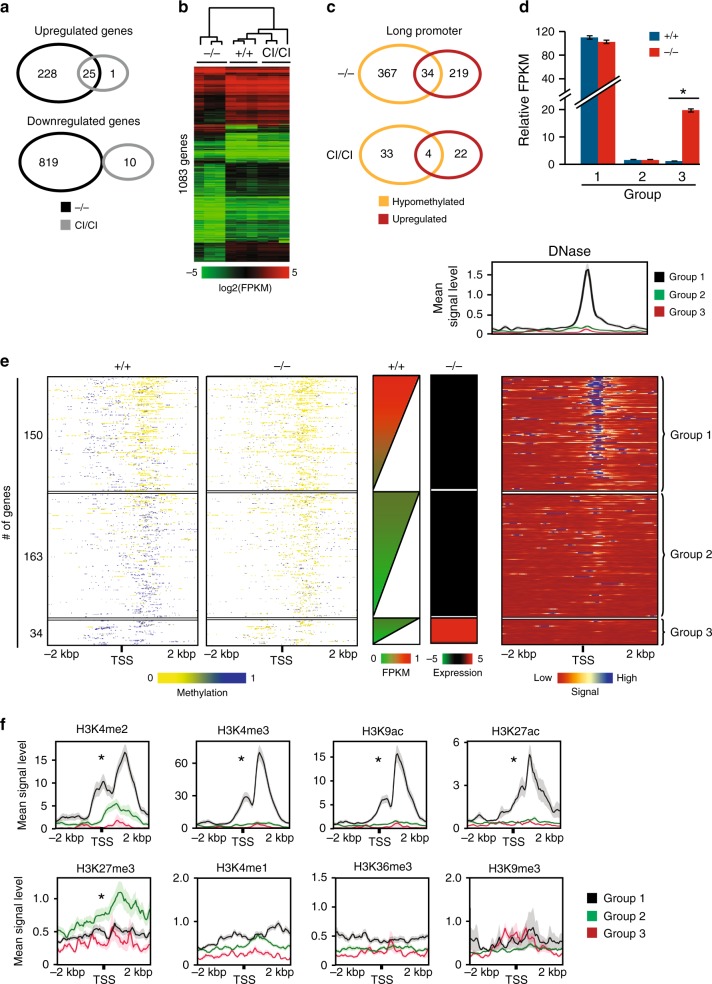
Absence of histone modifications is associated with methylation-dependent de-repression. **a** Weighted Venn diagrams displaying number of differentially expressed genes detected in *Dnmt3b*^*−/−*^ and *Dnmt3b*^*CI/CI*^ E11.5 embryos relative to *Dnmt3b*^*+/+*^ counterparts. *p* < 0.001 by hypergeometric test. **b** Heat maps of gene subset differentially expressed in embryos of indicated genotypes at E11.5 presented as log2(FPKM) values. Selection of 1083 genes was done by comparing expression values between *Dnmt3b*^*−/−*^ and *Dnmt3b*^*+/+*^ embryos (*n* = 3) (FC ≥ 2; *p* < 0.05 by DESeq). c Weighted Venn diagrams displaying overlaps between genes with hypomethylated DMRs and increased expression in *Dnmt3b*^*−/−*^ and *Dnmt3b*^*CI/CI*^ embryos relative to *Dnmt3b*^*+/+*^ counterparts; *p* < 0.001 by hypergeometric test. **d** Relative FPKM values ± SEM of genes with hypomethylated DMRs in long promoters with high FPKM in *Dnmt3b*^*+/+*^ and unchanged in *Dnmt3b*^*−/−*^ E11.5 embryos (Group 1, *n* = 150); low FPKM in *Dnmt3b*^*+/+*^ embryos and unchanged in *Dnmt3b*^*−/−*^ E11.5 embryos (Group 2, *n* = 163); and low FPKM in *Dnmt3b*^*+/+*^ embryos and increased expression in *Dnmt3b*^*−/−*^ (Group 3, *n* = 34). Data were normalized to mean FPKM values in Group 3 in *Dnmt3b*^*+/+*^ embryos. *(*p* < 0.01). Only genes with >2-fold expression increase were included. **e** Heat map summary of association of methylation and expression levels in *Dnmt3b*^*+/+*^ and *Dnmt3b*^*−/−*^ embryos in a region from −2 to +2 kbp around TSS of genes assigned to Groups 1–3. First two panels presents methylation levels of *Dnmt3b*^*+/+*^ and *Dnmt3b*^*−/−*^ embryos, third panel—FPKM values of *Dnmt3b*^*+/+*^ embryos, fourth—change in expression in *Dnmt3b*^*−/−*^ relative to *Dnmt3b*^*+/+*^ embryos, fifth panel presents results of DNAse I sensitivity assay (ENCODE); plot above shows mean signal ± SEM for groups 1, 2, and 3. Genes were sorted based on FPKM values in +/+ embryos. **f** Plots showing histone marks occupancy signals ± SEM in region from −2 to +2 kbp around TSS of genes in groups 1, 2, and 3; *p < 0.05 by two-tailed Student’s *t*-test. SEM values are presented as shading around mean value line. Source data are provided as a Source Data file

To analyze effects of Dnmt3b, we performed global gene expression RNA profiling from E11.5 WT, *Dnmt3b*^*−/−*^, and *Dnmt3b*^*CI/CI*^ embryos using RNA-sequencing. In total, 253 up-regulated and 819 downregulated genes (FC ≥ 2) were identified in *Dnmt3*^*−/−*^ embryos relative to WT controls (Fig. 3a). The most up-regulated genes were related to germ cells specific expression (e.g. *D1Pas1*, *Sohlh2*) while most downregulated genes included crystallins (e.g. *Cryga, Cryba4*) and matrilins (e.g. *Matn1, Matn4*). In addition, multiple poorly characterized protein coding genes such as *Gm43302*, *Gm43638*, *Gm37034* and non-coding RNA genes (*9330158H04Rik*; *Gm22009*) were also deregulated (Supplementary Data 3).

Like for DNA methylation, a vast majority of gene expression was rescued to the WT levels with only a subset of genes deregulated in *Dnmt3b*^*CI/CI*^ embryos (Fig. 3a). As a result, expression profiles of WT and *Dnmt3b*^*CI/CI*^ embryos clustered closer and apart of *Dnmt3b*^*−/−*^ (Fig. 3b, Supplementary Data 3).

We next analyzed association between DNA methylation and gene expression in various genomic elements. This analysis revealed that hypomethylation of ~9% (34/401) long promoters was associated with increased gene expression in *Dnmt3b*^*−/−*^embryos (Fig. 3c). Other elements, including gene bodies and enhancers, correlated with changes in methylation to a lower extent (Supplementary Fig. 9). Expression of few genes remained elevated in *Dnmt3b*^*CI/CI*^ embryos but this did not interfere with normal embryogenesis (Fig. 3a).

We next focused our analysis on the relationship between gene expression and hypomethylation in gene promoters. Out of 401 promoters methylated in WT, only 34 responded to hypomethylation by increased expression in E11.5 *Dnmt3b*^*−/−*^ embryos. We therefore asked whether other epigenetic changes already present in E11.5 WT embryos could predetermine increased expression upon hypomethylation in *Dnmt3b*^*−/−*^ embryos. To address this, we utilized genome-wide profiles of eight histone modifications generated from E11.5 WT embryos available at ENCODE (Bing Ren, UCSD lab, ENCSR231EPI, ENCSR215ZYV)^25^. By combining information on gene expression, DNA methylation, histone modifications, and chromatin accessibility, we identified three groups of genes with variable effects on expression in response to methylation loss.

The first group (Group 1, *n* = 150) is characterized by methylation upstream of TSS, high FPKM expression values, open chromatin around the TSS, and association with active histone marks (H3K4me2, H3K4me3, H3K9Ac, H3K27Ac) in E11.5 WT embryos (Fig. 3d–f, Supplementary Data 4). The second group (Group 2, *n* = 163) is characterized by methylation near the TSS, low FPKM expression values, closed chromatin, and association with repressive histone mark H3K27me3 in E11.5 WT embryos (Fig. 3d–f, Supplementary Data 4). In both Groups 1 and 2, loss of promoter methylation in E11.5 *Dnmt3b*^*−/−*^ embryos does not result in gene expression changes.

The third group (Group 3, *n* = 34) is characterized by methylation near the TSS, low FPKM expression values and closed chromatin, but no enrichment of chromatin marks in E11.5 WT embryos (Fig. 3d–f, Supplementary Data 4). Loss of promoter methylation in E11.5 *Dnmt3*^*−/−*^ embryos results in strong up-regulation of gene expression.

These results suggest that in a subset of promoters not enriched for any of the tested histone modifications, DNA methylation plays critical role as transcriptional inhibitor whereas in genes already expressed or marked by H3K27me3 its regulatory role is substantially lessened, and represents only one layer of regulation whose removal in *Dnmt3b*^*−/−*^ setting does not affect expression.

### Catalytic activity of Dnmt3b is associated with H3K27me3

**Fig. 4:**
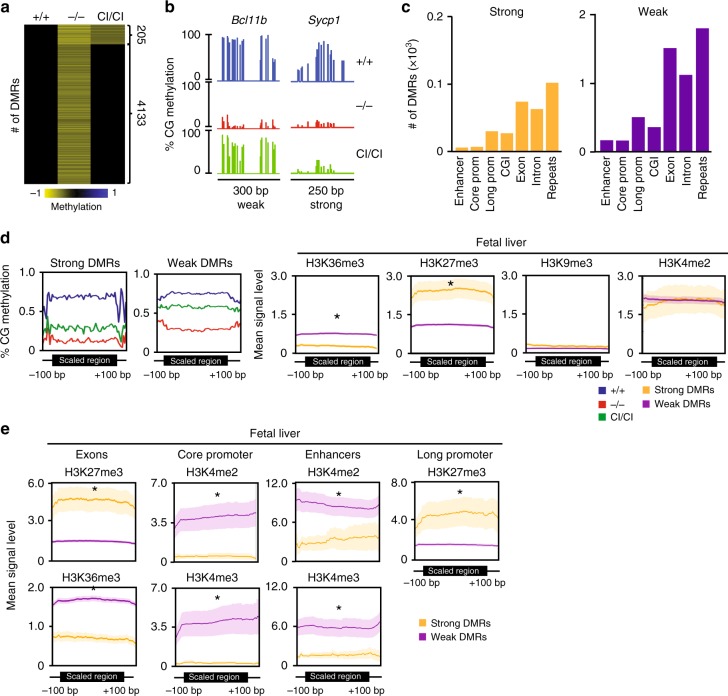
Association of histone modifications with Dnmt3b-dependent DNA methylation. **a** Differential requirement of DMRs on catalytic activity of Dnmt3b. Heat map presenting a subset of hypomethylated DMRs (*n* = 205) identified in E11.5 *Dnmt3b*^*−/−*^ relative to *Dnmt3b*^*+/+*^ depends strongly on catalytic activity of Dnmt3b because their hypomethylation is not rescued to wild-type levels in *Dnmt3b*^*CI/CI*^ E11.5 embryos (termed here strong). In contrast, DMRs hypomethylated in both E11.5 *Dnmt3b*^*−/−*^ and *Dnmt3b*^*CI/CI*^ relative to wild type depend on accessory rather than catalytic activity of Dnmt3b and are termed here *weak* (*n* = 4133). **b** DNA methylation scores obtained for *Bcll11b* (weak DMR) and *Sycp1* (strong DMR) from RRBS analysis of *Dnmt3b*^*+/+*^, *Dnmt3b*^*−/−*^ and *Dnmt3b*^*CI/CI*^ embryos at E11.5 as visualized by IGB. **c** Distribution of strong and weak DMRs among indicated genomic elements. **d** Enrichment of strong DMRs and weak DMRs for chromatin marks in E11.5 *Dnmt3b*^*+/+*^ embryos as detected by analysis of ChIP-seq (ENCODE). Averaged DNA methylation levels ± SEM for scaled regions of DMRs are shown for strong (*n* = 205) and weak DMRs (*n* = 4133) (first two panels). Mean levels ± SEM of four histone marks showing significant differences of enrichment between strong and weak DMRs are presented in the right panel. Plots shows profiles for DMRs scaled to same length and surrounding 100 bp unscaled regions. *p < 0.05 by two-tailed Student’s *t*-test. SEM values are presented as shading around mean value line. **e** Enrichment of strong and weak DMRs in enhancers, exons, core, and long promoters for significantly different chromatin marks in *Dnmt3b*^*+/+*^ embryos at E11.5 as detected by analysis of ChIP-seq from data obtained from ENCODE. Mean levels of histone marks showing significant differences of enrichment between strong and weak DMRs are presented. Plots shows profiles for DMRs scaled to same length and surrounding 100 bp unscaled regions. *p < 0.05 by two-tailed Student’s *t*-test. SEM values are presented as shading around mean value line. Source data are provided as a Source Data file

Our analysis revealed that 4133 DMRs hypomethylated in *Dnmt3b*^*−/−*^ embryos are similarly methylated between *Dnmt3b*^*+/+*^ and *Dnmt3b*^*CI/CI*^ DNA in E11.5 suggesting that their proper methylation primarily depends on the accessory function (Fig. 4a, Supplementary Data 5). These loci are weakly dependent on Dnmt3b’s CA and therefore we term them *weak*. An example of *weak* DMR is shown for the *Bcl11b* locus (Fig. 4b). In contrast, 205 loci remain largely hypomethylated in *Dnmt3b*^*CI/CI*^ embryos, suggesting that their methylation strongly depends on Dnmt3b’s CA (Fig. 4a, Supplementary Data 5) and we term them as *strong* (for example *Sycp1*; Fig. 4b). After annotating DMRs to genomic features, we found that both groups are relatively equally distributed across the genome with the majority of DMRs found in repetitive elements and gene bodies (Fig. 4c, Supplementary Fig. 10).

By comparing relative enrichments of histone marks in *Dnmt3b*^*+/+*^ embryo (ENCODE-ENCSR231EPI, ENCSR215ZYV)^25^ between genomic areas associated with strong and weak DMRs in *Dnmt3b*^*−/−*^ and *Dnmt3b*^*CI/CI*^ embryos, we found that weak DMRs are enriched in activating H3K36me3 when compared to the areas of strong DMRs (Fig. 4d). In contrast, strong DMRs are associated with higher levels of repressive mark H3K27me3 relative to *weak* DMRs (Fig. 4d). No significant differences in other histone marks were observed for weak and strong DMRs on a genome-wide level (Fig. 4d, Supplementary Fig. 11). However, further analysis of genomic elements revealed that the areas of weak DMRs are enriched for H3K36me3 only in exons but not in other parts of the genome. Weak DMRs are also associated with higher levels of H3K4me2 and H3K4me3 in core promoters and enhancers relative to strong DMRs (Fig. 4e). Strong DMRs are enriched in H3K27me3 in exons and long promoters. These associations were not limited to fetal liver but were seen also in neural tube (Supplementary Fig. 12).

Altogether, our data suggest that different mechanism may cover methylation of strong versus weak DMRs. For weak, Dnmt3b may serve as an accessory protein, whereas for strong its CA is irreplaceable.

Our results suggest different mode of action for Dnmt3b depending on whether catalytic or accessory activity is required. CA is favored by the presence of repressive H3K27me3 whereas accessory activity is promoted by the presence of activating marks such as H3K36me3 in exons and H3K4me2 and H3K4me3 in regulatory elements.

### Dnmt3b contributes to de novo methylation at E11.5

**Fig. 5:**
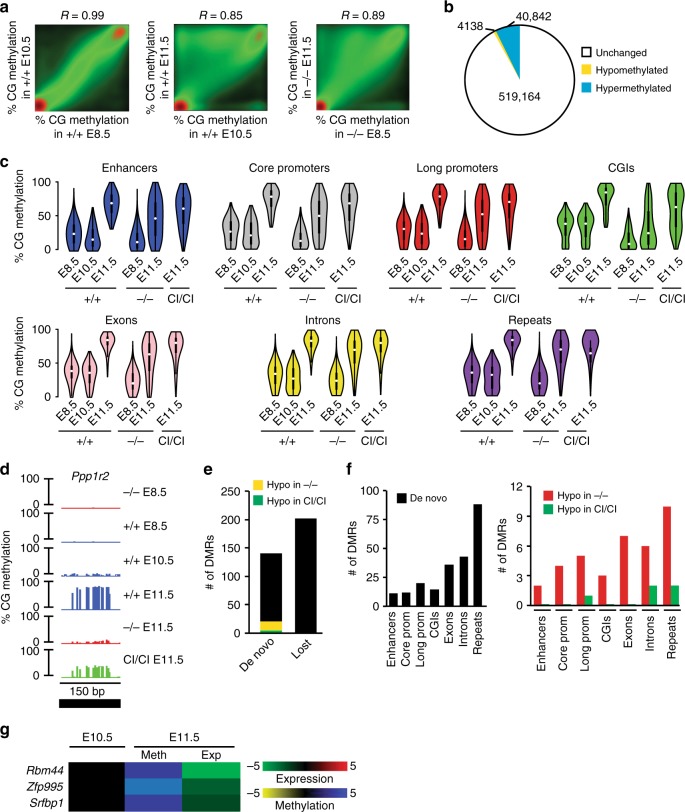
Methylation changes at E11.5 are partially mediated by Dnmt3b. **a** Pairwise comparison of CG methylation in *Dnmt3b*^*+/+*^ embryos at E8.5 and E10.5; E10.5 and E11.5 and *Dnmt3b*^*−/−*^ embryos at E8.5 and E11.5. The density of points increases from green to red. *R-*values represent Pearson correlation coefficients. **b** Total number of differentially methylated CGs in *Dnmt3b*^*+/+*^ embryos at E11.5 relative to E10.5 (meth diff. ≥30%). **c** Violin plots showing CG methylation in *Dnmt3b*^*+/+*^ embryos at E8.5, E10.5, and E11.5, *Dnmt3b*^*−/−*^ embryos at E8.5 and E11.5, and *Dnmt3b*^*CI/CI*^ embryos at E11.5 in indicated genomic elements. **d** Visualization of single-CpG profiles at de novo methylated locus of *Ppp1r2* that remains hypomethylated in *Dnmt3b*^*−/−*^ and *Dnmt3b* ^*CI/CI*^ embryos at E11.5. Visualization of single-CpG profiles at *Evc2* locus that maintains methylation across E8.5–E11.5 in *Dnmt3b*^*+/+*^ and loses methylation in *Dnmt3b*^*−/−*^. **e** The total number of hypermethylated (de novo) and hypomethylated (lost) DMRs identified in *Dnmt3b*^*+/+*^ embryos at E11.5 relative to E10.5 (*n* = 2 for each timepoint) (methylation diff. ≥ 30%; *p*(MWU) <0.05). Out of de novo methylated DMRs, those which are hypomethylated in *Dnmt3b*^*−/−*^ and *Dnmt3b*^*CI/CI*^ relative to *Dnmt3b*^*+/+*^ embryos at E11.5 are marked yellow and green, respectively. **f** Total number of hypermethylated (de novo) DMRs identified in *Dnmt3b*^*+/+*^ embryos at E11.5 relative to E10.5 within indicated genomic elements (left panel). Out of these, DMRs which are hypomethylated in *Dnmt3b*^*−/−*^ and *Dnmt3b* ^*CI/CI*^ embryos relative to *Dnmt3b*^*+/+*^ at E11.5 are marked red and green, respectively (right panel). **g** Heat map of genes de novo methylated within promoter or enhancers and downregulated (FC ≥ 2; *p* < 0.05 by DESeq) in *Dnmt3b*^*+/+*^ embryos at E11.5 relative to E10.5. Source data are provided as a Source Data file

After a wave of de novo methylation in early epiblast, the levels remain stable at least to E10.5, but whether any changes occur at E11.5 is unclear^13^. To address this, we first compared methylation of E8.5 and E10.5 WT embryos obtained previously^13^, using readouts of >5 × 10^5^ CGs (covered at ≥15*x* depth in all samples) and found no differences consistently with previous report^13^ (Fig. 5a). In contrast, we observed substantial gains and losses in CGs methylation between E10.5 and E11.5 WT embryos affecting various genomic elements (Fig. 5a–c, Supplementary Fig. 13). Methylation gains were partially suppressed in E11.5 *Dnmt3b*^*−/−*^ embryos, but mostly restored in *Dnmt3b*^*CI/CI*^ embryos suggesting that Dnmt3b plays de novo role in methylation between E10.5 and E11.5 (Fig. 5a–c, Supplementary Fig. 13). For example, the *Ppp1r2* locus is not methylated at E8.5, minimally at E10.5 and gains substantial methylation at E11.5 in WT but not in *Dnmt3b*^*−/−*^ embryos (Fig. 5d). Partial methylation is observed in *Dnmt3b*^*CI/CI*^ embryos although not to WT levels (Fig. 5d). Gain of methylation was observed even between *Dnmt3b*^*−/−*^ E8.5 and E11.5, suggesting involvement of other Dnmts in de novo methylation (Fig. 5a).

Further analysis revealed 133 hyper- and 196 hypomethylated DMRs between E10.5 and E11.5 *Dnmt3b*^*+/+*^ embryos (Fig. 5e, f). Out of these, gains in methylation of 18 DMRs in *Dnmt3b*^*+/+*^ embryos are likely dependent on Dnmt3b, as they are not present in *Dnmt3b*^*−/−*^ embryos (Fig. 5e, f). This dependence stems from an accessory rather than CA of Dnmt3b as the majority of gains are present also in *Dnmt3b*^*CI/CI*^ (Fig. 5e, f). Effects of methylation gains on gene expression were modest and were limited to 3/10 de novo methylated core promoters including *Rbm44*, *Zfp995,* and *Sfrfbp1* (Fig. 5g).

Despite prevalent increases in methylation, we identified more hypo- than hypermethylated DMRs affecting various genomic elements (Fig. 5e, Supplementary Fig. 14), suggesting that methylation gains are spread across genome while losses are localized to discrete regions of various genomic elements. Loss of methylation does not seem to affect gene expression and its relevance is unclear.

Altogether, our data identified changes in global methylation reflected by gains and losses in a subset of genomic elements and with moderate effects on transcription of few genes.

### Dnmt3b regulates major developmental pathways

The majority of *Dnmt3b*^*−/−*^ embryos die by age E12.5, suggesting that deregulated molecular events responsible for lethality accumulate by this stage of embryogenesis. To identify these events, we next utilized data obtained on gene expression profiles in *Dnmt3b*^*+/+*^, *Dnmt3b*^*−/−*^, and *Dnmt3b*^*CI/CI*^ embryos at day E11.5. Ingenuity pathway analysis (IPA) using 253 overexpressed and 819 underexpressed genes (FC ≥ 2) in *Dnmt3b*^*−/−*^ embryos relative to WT revealed multiple categories linked to embryogenesis.

**Fig. 6:**
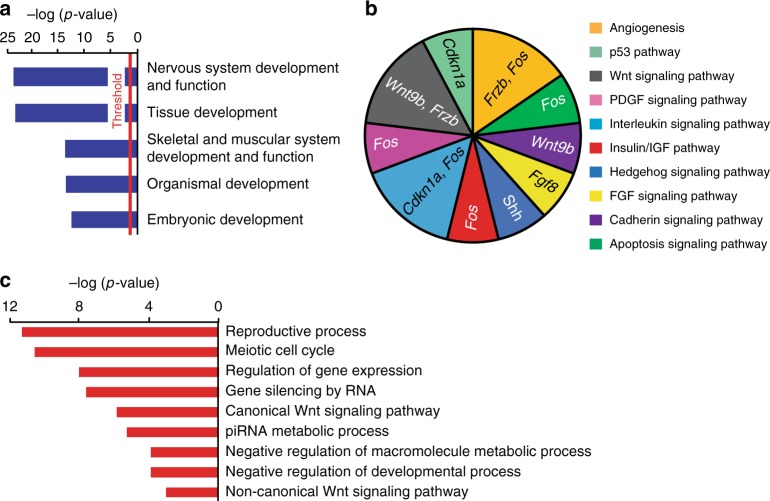
Dnmt3b controls developmental pathways involved in embryogenesis. **a** Ingenuity pathway analysis (IPA) of genes differentially expressed (FC ≥ 2, *p* < 0.05) in E11.5 *Dnmt3b*^*−/−*^ embryo. The top subcategories obtained in a category Physiological system development and function are displayed (*p* < 0.05 by right-tailed Fisher’s exact test). **b** Panther pathway analysis of upregulated genes (FC ≥ 2, *p* < 0.05 by DESeq) identified in E11.5 *Dnmt3b*^*−/−*^ embryo, which were found to be enriched in IPA analysis in a subcategory Embryonic development. **c** Gene ontology analysis of up-regulated genes (FC ≥ 2, *p* < 0.05 by DESeq) identified in E11.5 *Dnmt3b*^*−/−*^ embryo, which were found to be enriched in IPA analysis in a subcategory Embryonic development. Source data are provided as a Source Data file

For example, under the category Physiological system development and function, TOP five subcategories included Nervous system development and function, Tissue development, Skeletal and muscular system development and function, Organismal development, and Embryonic development underlining their link to development (Fig. 6a). Analysis of downregulated genes also identified deregulation of developmental pathways, such as Development of head, Formation of muscle, and Development of sensory organ (Supplementary Fig. 15).

Since Dnmt3b is a repressor, we reasoned that de-repressed rather than downregulated genes drive lethality in *Dnmt3b*^*−/−*^ embryos. Indeed, a deeper analysis of 28 genes specifically up-regulated in the subcategory Embryonic development revealed that these genes were involved in *Wnt signaling* (*Wnt9b, Frzb*), *FGF signaling* (*Fgf8*), *Hedgehog signaling* (*Shh*), *Angiogenesis* (*Frzb, Fos*), and *p53 pathway* (*Cdkn1a*) (Fig. 6b). The importance of Wnt signaling was further highlighted by Gene Ontology analysis that identified canonical as well as non-canonical signaling pathways as being activated (Fig. 6c, Supplementary Fig. 16).

Altogether, these data suggested that Dnmt3b may be directly involved in regulation of major developmental pathways including *Wnt* and *Hedgehog signaling*.

### Dnmt3b is a negative regulator of Wnt and hedgehog signaling

**Fig. 7:**
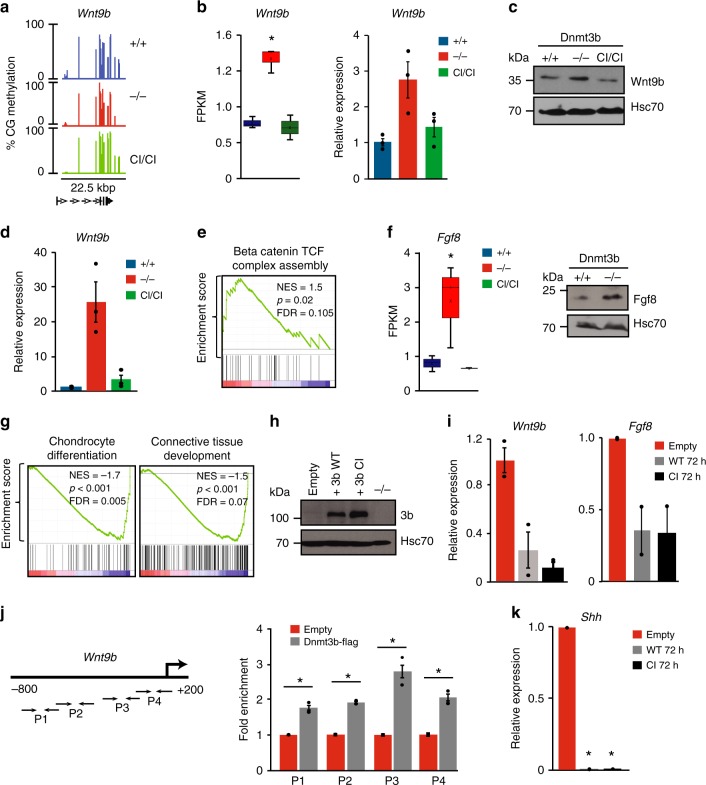
Dnmt3b represses *Wnt9b* and *Shh* in vivo and in vitro. **a** Methylation of *Wnt9b* locus in E11.5 embryos of indicated genotypes determined by RRBS. **b** *Wnt9b* expression by RNA-seq (*n* = 3; *p < 0.05 by DESeq; left) and real-time qRT-PCR (*n* = 3; normalized to *Gapdh*; right) in E11.5 embryos. Horizontal line represents median, bounds of box—likely range of variation and whiskers—min and max values. **c** Immunoblot of Wnt9b and Hsc70 levels in E11.5 embryos. **d** *Wnt9b* expression by real-time qRT-PCR in fetal brain of E11.5 embryos (*n* = 3), normalized to *Gapdh*. **e** GSEA using RNA-seq data shows positive enrichment in *Beta-catenin–TCF complex assembly* in E11.5 *Dnmt3b*^−*/−*^ embryos (*n* = 3). Normalized enrichment scores (NES), false discovery rate (FDR) and *p* values are shown. **f** Left. *Fgf8* expression by RNA-seq in *Dnmt3b*^*+/+*^, *Dnmt3b*^*−/−*^, and *Dnmt3b*^*CI/CI*^ E11.5 embryos (*n* = 3), *p < 0.05 (DESeq). Horizontal line represents median, bounds of box—likely range of variation and whiskers—min and max values. Right. Immunoblot analysis of Fgf8 levels in E11.5 embryos. **g** GSEA shows negative enrichment in developmental pathways in E11.5 *Dnmt3b*^*−/−*^ embryos relative to *Dnmt3b*^*+/+*^ (*n* = 3). **h** Dnmt3b and Hsc70 expression in mouse *Dnmt3b*^*−/−*^ T cell lymphoma cells (^*−/−*^) 72 h after transduction with lentiviruses expressing control vector (empty), Dnmt3b^WT^ (3b WT), Dnmt3b^CI^ (3b CI) as analyzed by immunoblot. **i** Real-time qRT-PCR analysis of *Wnt9b* and *Fgf8* expression in samples prepared as in **h**. Average of two independent experiments normalized to *β-actin*. **j** Left: Schematic of mouse *Wnt9b* promoter with position of primers (arrows): P1: −763/−582 bp, P2: −603/−411 bp; P3: −342/−127 bp; P4: −143/ + 36 bp relative to TSS. Right: Real-time qRT-PCR on DNA immunoprecipitated with anti-FLAG antibody from *Dnmt3b*^*−/−*^ lymphoma cells expressing FLAG tagged Dnmt3b^WT^ or empty vector. Data are shown as averaged fold enrichment over empty vector (*n* = 2), *p < 0.001 (two-tailed Student’s *t*-test). Assay was performed in triplicates and normalized to the input DNA. **k** Shh expression by real-time qRT-PCR in samples described in **h**. Averaged data of two independent experiments were normalized to *β-actin*, *p < 0.001 (two-tailed Student’s *t*-test). Data in figures **b**, **d**, **i**, **j,** and **k** are presented as means ± SEM. Source data are provided as a Source Data file

Analysis of deregulated expression in *Dnmt3b*^*−/−*^ embryos suggested that up-regulation of *Wnt9b* might be involved in their lethality. Next, we asked if any methylation changes are associated with regulatory elements including promoters, gene bodies, and enhancers that would explain elevated expression. This analysis revealed no measurable changes in 22.5 kbp region harboring *Wnt9b* gene or its putative enhancers among *Dnmt3b*^*+/+*^, *Dnmt3b*^*−/−*^, and *Dnmt3b*^*CI/CI*^ embryos at E11.5 (Fig. 7a, Supplementary Fig. 17). This suggested that *Wnt9b* may be regulated by Dnmt3b activities that are not directly linked to catalytic or accessory activity but rather repressive functions or, alternatively, by secondary events involved in embryonic death.

The expression of Wnt9b was significantly increased in E11.5 *Dnmt3b*^*−/−*^ embryos both at RNA and protein level consistently with RNA-seq data (Fig. 7b, c). This up-regulation was not observed in *Dnmt3b*^*CI/CI*^ embryos suggesting that presence of Dnmt3b^CI^ is sufficient for its normal physiological levels. Up-regulation of Wnt9b was more pronounced in fetal brain (Fig. 7d) and was also detectable in *Dnmt3b*^*−/−*^ E10.5 embryo (Supplementary Fig. 18), further highlighting its possible role in embryonic death of *Dnmt3b*^*−/−*^ mice. Consistently, β-catenin signaling signature was highly enriched in global gene expression data obtained from *Dnmt3b*^*−/−*^ embryos (Fig. 7e). Because Wnt9b was previously found to induce the expression of Fgf8 by binding of β-catenin to the transcription factor TCF^26^, we next analyzed expression of this genes specifically in E11.5 *Dnmt3b*^*−/−*^ embryos and found elevated levels (Fig. 7f). In addition, we observed negative enrichment of genes involved in developmental processes (Fig. 7g, Supplementary Fig. 19). This is consistent with previous findings^27^ that elevated Fgf8 inhibits a broad range of differentiation pathways including osteogenesis, chondrogenesis, neurogenesis, and myogenesis up to at least E14.5.

Because deregulated expression could be a consequence of impaired development, we next utilized *Dnmt3b*^*−/−*^ mouse lymphoma cells^21^ to test directly the effects of Dnmt3b on Wnt9b and Fgf8 expression. Importantly, exogenous expression of both Dnmt3b^WT^ and Dnmt3b^CI^ repressed *Wnt9b* and *Fgf8* (Fig. 7h, i), suggesting that Dnmt3b regulates these genes independently of CA. We next used chromatin immunoprecipitation (ChIP) assays to determine whether Dnmt3b could directly regulate the expression of Wnt9b. ChIP assays were performed using *Dnmt3b*^*−/−*^ mouse lymphoma cells^21^ overexpressing FLAG-tagged version of Dnmt3b^WT^. DNA immunoprecipitated with anti-FLAG antibody was amplified with primers flanking four regions of *Wnt9b* promoter (−800 to +200 bp) (Fig. 7j). The results from these ChIP assays showed enrichment of Dnmt3b in all tested regions especially in −342 to −127 bp region (Fig. 7j). Together, these results suggests that Dnmt3b directly represses Wnt9b independent of its CA.

Because enforced expression of Fgf8 and Wnt9b induces embryonic lethality in mice at E11.5–E15.5 (refs. ^27–29^), up-regulation of these genes likely contributes to lethality in *Dnmt3b*^*−/−*^ embryos at E11.5. The importance of repressor activity of Dnmt3b in mouse embryogenesis may be broader since both Dnmt3b^WT^ and Dnmt3b^CI^ also repressed expression of Shh, a key member of the *hedgehog* signaling pathway (Fig. 7k). This observation is consistent with Shh upregulated expression in *Dnmt3b*^*−/−*^ embryos at both E10.5 and E11.5 (Supplementary Fig. 18).

Altogether, our data suggest that Dnmt3b controls a wide range of developmental processes through Wnt9b, Fgf8, and *hedgehog* signaling pathways by its repressor activity that is crucial to prevent embryonic lethality.

## Discussion

In this study, we show that Dnmt3b is critical for embryogenesis by providing accessory function supporting catalytic activities performed by other Dnmts, as well as by exerting gene repression functions controlling essential developmental pathways. We further show that while its CA is dispensable for mouse embryogenesis it is irreplaceable for a subset of loci distributed across the genome suggesting double role for Dnmt3b in genome methylation. Finally, we show activities independent of the CA of Dnmt3b are linked to control of major developmental pathways including *Wnt* and *hedgehog* signaling. Thus, Dnmt3b is a multifaceted protein with several functions critical in mouse embryogenesis.

Dual roles of Dnmt3b in mediating methylation activities were recently reported in colorectal carcinoma cells in vitro^30^. Catalytically inactive Dnmt3b1 induced de novo methylation especially in gene bodies of actively transcribed genes in singly deficient DNMT1 or DNMT3B cells but not in DNMT3A/B double knockout cells. Such results suggested that Dnmt3b is an accessory protein for Dnmt3a mediated enzymatic activities.

We discovered dual capacity of Dnmt3b in embryogenesis described here by comparing methylation in WT, *Dnmt3b*^*−/−*^ and *Dnmt3b*^*CI/CI*^ E11.5 embryos. Accessory activity of Dnmt3b was manifested by loci hypomethylated in *Dnmt3b*^*−/−*^, but not in *Dnmt3b*^*CI/CI*^ embryos. In contrast, strong dependence on intrinsic Dnmt3b’s CA was manifested by loci hypomethylated in both genetic settings relative to controls.

Surprisingly, the accessory function of Dnmt3b seems broader in scope than its catalytic function, as methylated loci associated with such activity represent 95% of identified DMRs in embryogenesis. Association with different histone marks in the WT setting further highlights possible differences in biology of these regions.

In exons, the targets of accessory activity are enriched in activating H3K36me3 unlike targets of CA. Preferential binding of DNMT3B to regions enriched for H3K36me3 modification was previously reported, suggesting that DNA methylation might be guided to chromatin carrying this mark^11,31^. In exonic areas, Dnmt3b accessory function may link H3K36me3 to other methyltransferases such as Dnmt3a or Dnmt1 that mediate DNA methylation. In such scenario Dnmt3b would function similarly as catalytically inactive Dnmt3L that links Dnmt3a/b to unmethylated H3K4 (ref. ^8^). Consistent with such idea is our observation that Dnmt3L is up-regulated in *Dnmt3b*^*−/−*^ but not *Dnmt3b*^*CI/CI*^ embryos at E11.5, perhaps due to attempt to compensate for loss of Dnmt3b’s accessory function. Targets of Dnmt3b’s accessory function in other parts of the genome are not associated with enrichment in H3K36me3 and therefore likely involving different histone marks. This is supported by observation of H3K4me2 and H3K4me3 enrichments in long promoters and enhancers relative to areas dependent on catalytic activity. Alternatively, Dnmt3b contributes to stabilization and activation of complexes between DNMT3L and other DNMTs. Indeed, inactive Dnmt3b isoform was shown to stabilize and increase activity of Dnmt3a-Dnmt3L in vitro^16^. While precise mechanism needs to be determined, it is clear that Dnmt3b plays a major accessory role in embryogenesis similar to Dnmt3L.

Previous studies clearly established that Dnmt3L is required for gametic methylation and fertility. Unlike *Dnmt3b*^*CI/CI*^ mice, *Dnmt3L*^*−/−*^ mice show impairment in sperm maturation and oocytes methylation resulting in sterility^8,32,33^. However, this deficiency in vivo does not compromise somatic methylation, as *Dnmt3L*^*−/−*^ mice are viable and display normal methylation patterns in tissues, suggesting that Dnmt3L is not the only accessory protein in embryogenesis^8,34,35^. Additionally, Dnmt3L does not bind directly to H3K36me3 (ref. ^36^), a major histone modification associated with regions rescued through methylation-independent properties of Dnmt3b. It is therefore possible that one role of Dnmt3b is to provide accessory functions in embryogenesis either throughout embryonic development or after E8.5 when levels of Dnmt3L are decreasing.

Consistent with such idea is the observation that *Dnmt3b*^*−/−*^ embryos die at mid-gestation^5,14^. It remains unanswered which of the Dnmts is recruited by Dnmt3b’s accessory function to provide enzymatic activity to targeted loci. While most likely candidate appears to be Dnmt3a, this enzyme is dispensable for embryogenesis^5^. Consequently, the role of Dnmt1 as an enzyme providing catalytic activity cannot be ruled out, Dnmt1 clearly plays a critical task in embryogenesis as *Dnmt1*^*−/−*^ embryos die at E9.5.

In contrast to accessory activity, targets of CA are associated with higher levels of repressive mark H3K27me3 in exons and long promoters. Although DNA methylation and H3K27me3 are typically mutually exclusive, it was previously shown that Dnmt3b can engage targets with H3K27me3 and methylate the surrounding DNA^2^. Thus, at least in some genomic areas Dnmt3b executes CA. However, the molecular basis and physiological role of this phenomenon needs further investigation especially because our analysis of histone marks is limited to the wild-type setting.

Another interesting aspect of this study comes from analysis of histone modifications of genes with promoter hypermethylation in WT but hypomethylation in *Dnmt3b*^*−/−*^ E11.5 embryos. Genes associated with open chromatin, active histone marks, and transcription in WT setting did not enhance expression in response to loss of methylation in *Dnmt3b*^*−/−*^ E11.5 embryos. Similarly, loss of methylation did not affect expression of genes associated with closed chromatin and with repressive mark H3K27me3. However, a subset of 34 genes associated with closed chromatin and silencing were strongly up-regulated in *Dnmt3b*^*−/−*^ embryos. Interestingly, promoters of these genes were not enriched in any histone mark in *Dnmt3b*^*+/+*^ embryos. Thus, in this group, DNA methylation serves as a strong inhibitor of transcription and seems to be a sole determinant whether gene becomes expressed upon loss of DNA methylation. Some of these genes have been previously identified as dependent solely on methylation for their expression, such as *Dazl, Piwil2*, and *Asz1* and being involved in genome defense against transposable elements during the germline cell development^37^. Thus, our data support the idea that certain parts of the genome contain chromatin lacking repressive or activating histone modifications, in which DNA methylation is a key regulatory mechanism of expression^37^.

Our studies also revealed deregulation of pathways potentially involved in embryonic lethality of *Dnmt3b*^*−/−*^ mice, including *Wnt* and *hedgehog* signaling. We detected up-regulation of Wnt9b that coincides with morphological defects especially observed in the heart of E11.5 *Dnmt3b*^*−/−*^ embryos. Importantly, Dnmt3b^WT^ is recruited to Wnt9b promoter and represses expression in vitro, suggesting that Wnt9b is a direct target of Dnmt3b. Similarly, Dnmt3b^CI^ repressed Wnt9b demonstrating that such effect is independent of MT activity. Whether this repression is mediated through recruitment of HDACs, polycomb complexes, or other mediators remains to be seen^38,39^.

*Wnt* signaling is involved in all aspects of embryogenesis and ectopic overexpression of Wnt9b leads to embryonic death at E11.5 to 12.5 primarily due to cardiac insufficiency and hemorrhage^28^. Timing when such up-regulation exerts lethal consequences in embryogenesis and manifestation of similar morphological defects strongly support the idea that up-regulation of Wnt9b contributes to lethality of *Dnmt3b*^*−/−*^ embryos. This is further supported by our observations that Fgf8 is up-regulated in *Dnmt3b*^*−/−*^ embryos and ectopic expression of Dnmt3b^WT^ and Dnmt3b^CI^ Dnmt3b represses Fgf8 expression in *Dnmt3b*^*−/−*^ cells in vitro. Fgf8 is activated by Wnt9b and its overexpression in CNC leads to craniofacial defects and embryonic lethality by E14.5 due to disrupted cell differentiation and organogenesis^26,27,29,40^. Hence, up-regulation of Wnt9b and Fgf8 is contributing to embryonic death.

However, increased *Wnt* signaling may not be the only event involved in lethality of *Dnmt3b*^*−/−*^ embryos. We also observed activation of *hedgehog* signaling through upregulation of *Shh* in *Dnmt3b*^*−/−*^ embryos. Like for Wnt9b, Shh expression is strongly repressed by overexpression of Dnmt3b^WT^and Dnmt3b^CI^ in *Dnmt3b*^*−/−*^ cells in vitro suggesting involvement of Dnmt3b in its regulation. *Shh* is a crucial regulator of development including ventral patterning of the neural tube brain and heart^41,42^. Its overexpression in transgenic mice leads to craniorachischisis with major alterations in long bones and death at birth^43^.

Lastly, in this study we also identified E11.5 as a developmental stage at which measurable methylation changes occur. Transition from E8.5 to E10.5 is characterized by stable methylation patterns with minimal changes^13^. In contrast, modest methylation gains and losses occur from E10.5 to E11.5 partially in Dnmt3b-dependent manner.

Both gains and losses affected all genomic areas but losses affected more repetitive elements and gene bodies. While modest in scope, gains in promoter methylation are associated with decreased expression of several genes including *Rbm44, Zfp995* and *Srfbp1*. Thus, E11.5 day of embryogenesis may be marking the beginning of more robust changes accumulated as results of acquisition of tissue-specific methylation patterns.

## Methods

### Cell cultures, lentiviruses, and FACS

Lenti-XTM 293 T Cell Line was purchased from Takara (Clontech). Mouse Dnmt3b^*−/−*^ cell line was established as described before^21^. Lentiviral vectors pLV-mDnmt3b-EF1A-mCherry, pLV-mDnmt3b-FLAG-EF1A-mCherry, pLV-mDnmt3b (P705V, C706D)-EF1A-mCherry, and pLV-EF1A-mCherry were obtained from VectorBuilder. Cells were maintained in DMEM or RPMI 1640 (Invitrogen) containing 10% fetal bovine serum. Cell lines were cultured at 37 °C in a humidified 5% CO_2_ atmosphere and were passaged according to recommendations.

To generate lentiviruses, 293T cells were seeded in 10-cm tissue culture plate to obtain ~80–90% confluence and transfected with construct and packaging plasmids psPAX2 and pMD2.G at ratio 1:0.65:0.35 using 70 μg of polyethylenimine (PEI) (Polysciences). Virus was collected 48–96 h post transfection. Transduction was performed as described previously^21^, using either *Dnmt3b*^*−/−*^ or *Dnmt3a*^*−/−*^*;Dnmt3b*^*−/−*^ T cell lymphoma lines^20,21^. Efficiency of transduction was determined by measuring percentage of mCherry + cells by FACS (Supplementary Fig. 20). Transduced cells were harvested 72–96 h after transduction

For in vitro studies (Fig. 7h, i, k), Dnmt3b^WT^, Dnmt3b^CI^, and empty vector overexpressing *Dnmt3b*^*−/−*^ lymphoma cells were harvested at 72 h after transduction and used for RNA extraction using standard methods.

For ChIP assay (Fig. 7j), FLAG-tagged Dnmt3b^WT^-overexpressing *Dnmt3b*^*−/−*^ lymphoma cells were collected at 72 h after transduction, and cells were immediately processed for ChIP.

For RRBS analysis (Supplementary Fig. 2), Dnmt3b^WT^, Dnmt3b^CI^, and empty vector overexpressing *Dnmt3a*^*−/−*^*;Dnmt3b*^*−/−*^ lymphoma cells were harvested 96 h post transduction and cells were used for DNA extraction using phenol/chloroform extraction and ethanol precipitation.

### Mouse studies

Mice harboring conventional knock-in mutations (P656V and C657D) in Dnmt3b coding sequence (*Dnmt3b*^*C*I^) were generated by microinjection of CRISPR/Cas9 and oligo donor in fertilized eggs independently in FVB and C57BL/6 backgrounds. The mutations (CCATGC >GTCGAC) in donor oligo were introduced into exon 19 of Dnmt3b gene (NM_001003961.4) by homology-directed repair. A silent mutation (GTG to GTC) was also introduced upstream to prevent the binding and re-cutting of the sequence after homology-directed repair. Cas9 mRNA, sgRNA, and donor oligo were co-injected into zygotes for knock-in mouse production. The pups were genotyped by PCR, followed by sequence analysis. Positive founders were bred to generate *Dnmt3b*^*+/CI*^ mice used in these studies. PCR-based genotyping of genomic DNA isolated from the tails was used to confirm genotypes. Sequences of F1, F2, R primers are in Supplementary Data 6. The offspring of two independent mouse lines for each genetic background were analyzed. No phenotypic differences were observed in behavior of founder-specific offspring. All experimental animal procedures were approved by the Institutional Animal Care and Use Committee (IACUC) at the University of Florida under protocol number 201609589 and complied with all relevant ethical regulations for animal testing and research. All mice were housed in a pathogen-free barrier facility at the UF.

All embryos were obtained from naturally mated *Dnmt3b*^*+/−*^ and *Dnmt3b*^*+/CI*^ mice. The morning on which the vaginal plug was observed was designed E0.5 days. Whole embryos were dissected at E10.5, E11.5, or E12.5 and examined under a microscope. Viability of embryos was assessed by detecting the heart beat and blood supply to the yolk sac. Only live embryos were processed for further analysis. Digital images of embryos were captured with a Zeiss Stemi 305 CAM Digital Stereo Zoom Microscope (Carl Zeiss, Inc., Thornwood, NY).

Genomic DNA was prepared by proteinase K digestion followed by phenol/chloroform extraction and ethanol precipitation. DNA isolated from yolk sac was taken for genotyping. Total RNA was isolated as previously described^20^ from *Dnmt3b*^*+/+*^; *Dnmt3b*^*−/−*^ and *Dnmt3b*^*CI/CI*^ embryos.

To generate conventional Dnmt3b knockout allele, we used approach described previously^21,44,45^ utilizing Dnmt3b^2loxP^ mice obtained from E. Li (Novartis Institutes for Biomedical Research, Cambridge, Massachusetts, USA). we generated *Dnmt3a*^*−/−*^*;Dnmt3b*^*−/−*^ cell line previously^20^. Mice were kept in FVB/N genetic background.

### Chromatin immunoprecipitation

Mouse *Dnmt3b*^*−/−*^ lymphoma cells (1 × 10^7^) transduced with empty or Dnmt3b-FLAG expressing vector were crosslinked in 1% formaldehyde and quenched with 0.125 M glycine. Cells were washed twice with cold PBS and then resuspended in cell lysis buffer (10 mM Tris-HCl pH 7.5, 10 mM NaCl, 3 mM MgCl_2_, 0.4% NP-40) to extract nuclei. Nuclei were lysed by incubating in 100 µL SDS lysis buffer (1% SDS, 10 mM EDTA, 50 mM Tris-HCl pH 8.0, protease inhibitors) on ice for 10 min. DNA was sonicated to yield 200–500 bp chromatin fragments, and cleared by centrifugation. The supernatant was diluted in ChIP buffer (0.01% SDS, 1% Triton X-100, 1 mM EDTA, 20 mM Tris-HCl pH 8.0, 150 mM NaCl, and protease inhibitors). The supernatant containing sheared chromatin was precleared by protein G Dynabeads (Invitrogen; 10004D) and the supernatant was incubated with 10 µg of anti-FLAG antibody (Sigma; F3165) overnight at 4 °C with gentle rotation. Protein G Dynabeads were added and the mixture was rotated for 2 h at 4 °C. DNA was purified by phenol/chloroform/isoamyl alcohol and subsequent chloroform extractions and ethanol precipitation. Isolated DNA was used in RT-qPCR analysis to measure levels of enrichment of Wnt9b promoter region in cells expressing FLAG-Dnmt3b. Primer sequences for qPCR are provided in Supplementary Data 6.

### Histology

Formalin-fixed paraffin-embeded sections (4 µm) of whole emrbyos were stained with hematoxylin (Sigma, H9627) for 40 s and with eosin (Sigma-Aldrich, HT110116) for 30 s. The tissue sections were mounted with Permount mounting medium (Fisher Scientific, SP15-100). All procedures were conducted by Molecular Pathology Core, University of Florida. Images were generated with a Zeiss Axio Imager 2 microscope (Carl Zeiss, Inc., Thornwood, NY).

### Western blotting

Western blots were performed as previously described^21^, using the following antibodies: Dnmt1 (ab188453, Abcam; dilution 1:5000), Dnmt3a (SC-20703, Santa Cruz; dilution 1:1000), Dnmt3b (PA1-884, Thermo Fisher; dilution 1:1000), Dnmt3L (SC-393603, Santa Cruz; dilution 1:1000), Fgf8 (MAB323, R&D Systems; dilution 1:1000), Wnt9b (AF3669, R&D Systems; dilution 1:1000), Hsc-70 (SC-7298, Santa Cruz; dilution 1:10,000). Uncropped and unprocessed scans of blots are available in Source Data file.

### RRBS and bioinformatics analysis

The RRBS libraries were prepared and sequenced at the Medical Genome Facility at the Mayo Clinic, Rochester and ran on an Illumina HiSeq2500 sequencer. Publicly available RRBS data for *Dnmt3b*^*+/+*^ and *Dnmt3b*^*−/−*^ embryos were obtained from GEO (GSE60334)^13^. Quality check, trimming, filtering, and alignment of reads to the reference genome was performed at the ICBR Bioinformatics Core (UF, Florida). The aligned BAM files were uploaded to the Galaxy web platform^46^. Methylation calling was performed with Methyldackel (v 0.3.0.1) using the mm10-CG index (https://github.com/dpryan79/MethylDackel.git). For methylation analysis in embryos, only CpG sites with a minimum sequencing depth 15× were included in analysis. For methylation analysis *in Dnmt3b*^*−/−*^*;Dnmt3a*^*−/−*^ lymphoma cells overexpressing Dnmt3b^WT^, Dnmt3b^CI^, and empty vector, only CpG sites with a minimum sequencing depth of 10× were included in analysis. Methylation scores were visualized with the Integrated Genome Browser (IGB)^47^. Scatter plots of methylation score were generated in Rstudio v1.1.4.6 using package gplots^48^ and violin plots were generated in BoxplotR^49^. Genome-wide Pearson correlation analysis of CpG sites was performed using deepTools package multiBigWigsummary and plotCorrelation^50^.

DMRs were determined by Metilene^51^ and defined based on average of minimum three consecutive DMCs with methylation change of ≥30 percent in the same direction with *p* values <0.05 (as determined by MWU test). Maximal base pair cut-off for a distance between consecutive DMCs in DMR was set to 50 or 100 bp for embryonic or *Dnmt3b*^*−/−*^*;Dnmt3a*^*−/−*^ lymphoma data, respectively.

Annotation of methylated CpGs and DMRs to long promoters, core promoters, gene bodies, exons, introns, CGIs, enhancers, and repeats was performed using bedtools intersect. The DMR was retained if the overlap between these elements and DMR was at least 50% of the length of the DMR. Chromosomal coordinates of TSS, gene bodies, exons, introns, CGIs, and repeats were acquired from the USCS Table browser. Enhancers coordinates identified in forebrain, hindbrain, heart, limb, and hindlimb bud at E11.5 were obtained from Enhancer atlas^52^. Long promoter was defined as 1500 bp upstream to 500 bp downstream of the TSS. Core promoter was defined as 300 bp upstream to 150 bp downstream of the TSS. Gene body was defined as 500 bp downstream of the TSS up to the end of transcript. Hierarchical clustering of CpG methylation percentages within DMRs was performed using Cluster 3.0 (ref. ^53^) and heat maps were visualized in Java TreeView 3.0 (ref. ^54^). DeepTools^50^ was used to display traces and heat maps.

### ChIP-seq data analysis

Publicly available data on chromatin modifications in Dnmt3b^+/+^ embryos at E11.5 were obtained from ENCODE (Bing Ren, UCSD lab, ENCSR231EPI, ENCSR215ZYV)^25^. Heat map of DNAse I sensitivity assay and profiles of peak signals were generated using the computeMatrix, plotProfile and plotHeatmap scripts from the deepTools3 package^50^. Plots showing histone enrichment profiles across DMRs scaled to 1000 bp were computed using 10 bp long non-overlaping bins. Flanking un-scaled −100 and +100 bp regions are shown. Plots presenting histone modification profiles for genes with hypomethylated promoters that become up-regulated or retain their expression in *Dnmt3b*^*−/−*^ embryos show unscaled region from −2 to +2 kbp around TSS.

### Combined bisulfite restriction analysis

Combined bisulfite restriction analysis (COBRA) was carried out as described previously^55^. Briefly, bisulfite conversion of genomic DNA was carried out using the Epitect Bisulfite Kit (Qiagen). PCR products were digested with restriction enzymes *Bst*UI and *Taq*I (NEB). Digested products were then loaded on an 8% PAGE gel, separated by electrophoresis, and stained by SYBR Gold (Invitrogen).

Mouse bisulfite specific primers are shown in Supplementary Data 6.

### Bisulfite sequencing

Bisulfite conversion of genomic DNA was carried out using the Epitect Bisulfite Kit (Qiagen). PCR fragments from bisulfite treated DNA were cloned using pGem Easy T-cloning kit (Promega). Plasmid DNA isolated from individual clones was sequenced by Sanger sequencing (Genewiz). Mouse bisulfite specific primers are shown in Supplementary Data 6.

### Quantification of 5mdCs

Combined liquid chromatography-tandem mass spectrometry with multiple reaction monitoring (LC-MS/MS-MRM) was applied to quantify 5dmCs levels in DNA extracted from mouse *Dnmt3b*^*+/+*^; *Dnmt3b*^*−/−*^ and *Dnmt3b*^*CI/CI*^ E11.5 embryos^56^. DNA hydrolysis was performed by using DNA Degradase Plus (Zymo Research). Briefly, 400 ng of genomic DNA was mixed with 2 µL of 10× DNA Degradase Reaction buffer, 1 µL DNA Degradase Plus and water to make a total reaction volume of 20 µL. Finally, the reaction was inactivated heating to 70 °C for 20 min. Liquid phase chromatography was performed using gradient elution from a 2.1 × 100 mm Hypersil GOLD aQ C18 reversed-phase column with a guard cartridge and an Accela 600 quaternary pump at 250 µL/min at 40 °C. Transition pairs (258.2 → 141.93*m*/*z* for 5HmdC, 242.2 → 126.10*m*/*z* for 5mdC, and 268.1 → 152.3*m*/*z* for dG) were monitored on a Thermo Scientific TSQ Vantage Triple Quadrupole mass spectrometer at dwell times of 0.375 s each and scan widths of 0.002*m*/*z*. The collisional gas pressure was set at 1.2 mTorr. The raw data were analyzed with the Xcalibur Qual Browser using ICIS peak detection with following parameters: baseline windows of 30 units for 5mdC and 5HmdC, windows of 40 units for dG, the minimum signal-to-noise ratio 3.0, and the peak detection set for the highest peak option. Concentrations of 5mdC were calculated as a percentage of 2′-deoxyguanosine (dG). The calibrated ranges for the samples were 0–25% for 5mdC using a fixed 40 pmol amount of dG as an internal standard. Replicates for the unknown samples were run in triplicate followed by a blank to eliminate carryover into the next unknown run. All the procedures were carried out by Zymo Research Epigenetic Services (Zymo Research Corp).

### Real-time qRT-PCR

RNAs were reverse transcribed with the SuperScript III Reverese transcriptase (Thermo Fisher) using oligo(dT) primers. Real-time qRT-PCR was performed with the iQ™ SYBR® Green Supermix (Bio-Rad) on a CFX96 Touch™ Real-Time PCR Detection System (Bio-Rad). Fast PCR cycling conditions were used (95 °C for 3 min, 40 cycles (95 °C for 10 s, 58–63.5 °C for 30 s)), followed by a dissociation curve analysis. All qPCR measurements were performed in duplicate reactions and normalized to the expression of three housekeeping genes (*Rpl4*, *β-actin, Gapdh*). In parallel, no-RT controls were amplified to rule out the presence of contaminating genomic DNA. Primer sequences for qPCR are provided in Supplementary Data 6.

### RNA-seq

Library generation and sequencing was performed on NovaSeq 6000 platform using paired-end 150 bp runs (Novogene, USA).

Publicly available RNA-seq data for *Dnmt3b*^*+/+*^ at E10.5 were obtained from GEO (GSE47033) ^57^. Trimmed sequencing data were first aligned to *Mus musculus* UCSC mm10 reference genome using STAR aligner^58^. RNA-seq data with minimum mapped quality 50 were quantitated using the RNA-seq quantitation pipeline in SeqMonk software (http://www.bioinformatics.babraham.ac.uk/projects/seqmonk/). DeSeq2 was used to calculate differential expression ^59^. For differentially expressed genes, only genes with a fold change ≥2 and a *p* value <0.05 were considered to be significant. Hierarchical clustering was performed in Cluster 3.0 (ref. ^53^) and heat maps were visualized in Java TreeView 3.0 (ref. ^54^). Gene ontology enrichment analysis was conducted using the Gorilla software^60^. Pathway enrichment was performed using Panther pathway analysis^61,62^.

### Ingenuity pathway analysis

Differentially expressed genes (FC ≥ 2, *p* < 0.05), which were found in *Dnmt3b*^*−/−*^ embryos when compared to *Dnmt3b*^*+/+*^ embryos and were rescued in *Dnmt3b*^*CI/CI*^ embryos, were analyzed using IPA (Qiagen)^63^. Core analysis was performed to identify top ranking pathways and categories for differentially expressed genes. Top five categories obtained in Physiological system development and function were displayed (*p* < 0.05, for all subcategories).

### Gene set enrichment analysis

All FPKM values for *Dnmt3b*^*−/−*^, *Dnmt3b*^*CI/CI*^ and *Dnmt3b*^+/+^ embryos were converted to GCT expression dataset. CLS files were generated using CLSFileCreator (v4) (http://software.broadinstitute.org/cancer/software/genepattern/modules/docs/ClsFileCreator/4). Hallmark Signature gene sets, Curated gene sets, and GO gene sets were downloaded from Broad Institute’s Molecular Signatures Database. Gene Set Enrichment Analysis (v3.0)^64,65^ was used to test the relationship between RNA-seq expression data and the Hallmark Signature gene sets, Curated gene sets and GO gene sets, which were downloaded from Broad Institute’s Molecular Signatures Database. Gene sets enriched in less than 15 genes and more than 500 genes were excluded from the analysis. Gene sets with a false discovery rate (FDR) value <0.25 and *p* < 0.05 after performing 1000 permutations were considered to be significantly enriched.

### Statistical analysis

Statistical significance of means ± SEM were evaluated using the two-tailed Welch’s *t*-test. For all statistical analyses *p* values <0.05 were considered significant. The significance between observed and expected genotype representation of embryos was calculated using Chi-squared test. The significance of the overlapping genes was calculated in R version 3.4.3 using the hypergeometric distribution function phyper. Differential histone enrichment between weak and strong DMRs was analyzed by Student’s *t*-test or Welch’s unequal variances *t*-test with threshold of *p* value <0.05. Clustering was performed on the basis of average linkage hierarchical uncentered clustering of the genes.

### Reporting summary

Further information on research design is available in the Nature Research Reporting Summary linked to this article.

## Supplementary information

Supplementary Information

Reporting Summary

Peer Review File

Description of Additional Supplementary Files

Supplementary Data 1

Supplementary Data 2

Supplementary Data 3

Supplementary Data 4

Supplementary Data 5

Supplementary Data 6

Source Data

## Data Availability

The RRBS and RNA-seq data are deposited at the NCBI Gene Expression Omnibus database^66^ under the number GSE132952. All other relevant data supporting the key findings of this study are available within the article and its Supplementary Information files or from the corresponding author upon reasonable request. The source data underlying Figs. 1e, f, i, j, 3d, 7b, c, d, f, h, i, j, k are provided as a Source Data file. A reporting summary for this article is available as a Supplementary Information file.
