# Supplementary material for: Catalytically inactive Dnmt3b rescues mouse embryonic development by accessory and repressive functions
Source: Nat Commun. 2019 Sep 26;10:4374. doi: 10.1038/s41467-019-12355-7 (PMC6763448; doi:10.1038/s41467-019-12355-7)
Supplement: Supplementary file 1 — Supplementary Information [file 41467_2019_12355_MOESM1_ESM.pdf]

a

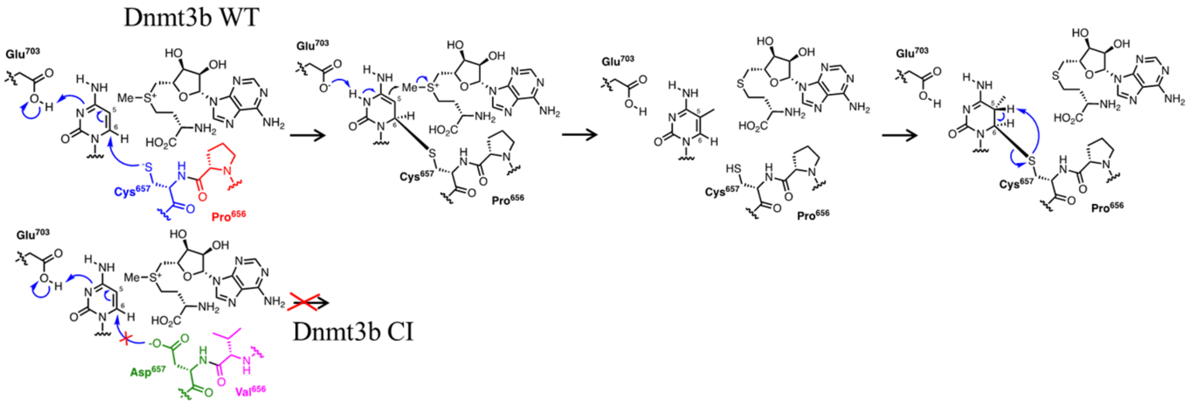

b

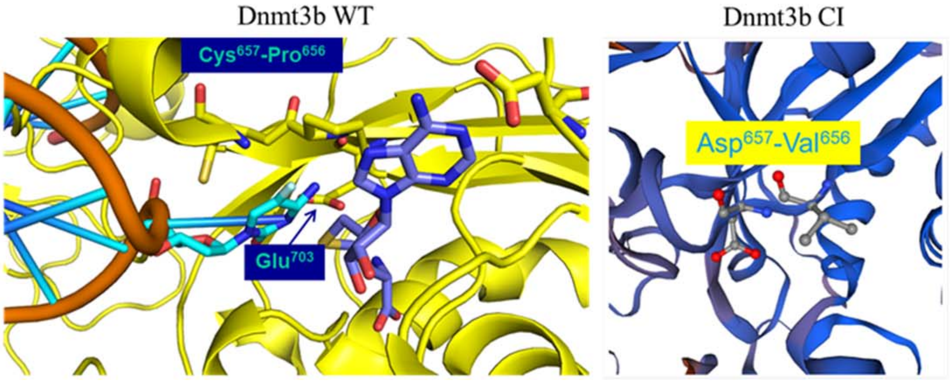

Suplementary Fig. 1. Rationale for the double amino acid substitution to inactivate catalytic function of Dnmt3b.

a A schematic describing the molecular basis for the catalytic function generated using ChemDraw Professional 16.0.

b The coordinates from 4U7P and 1MHT were overlayed using PyMol v1.8. *Left:* The *wild-type* Dnmt3b is shown as yellow cartoon with the key residues (Glu703, Cys657 and Pro656) shown as stick, SAM is shown in purple and the DNA fragment is shown in orange and cyan. Cys657 is the residue that catalyzes the methyltransferase reaction and Pro656 creates the pocket that holds the SAM in place to facilitate the transfer. *Right:* The catalytically inactive Dnmt3b (blue) in which Cys657 and Pro656 were substituted with Asp657 and Val656 (gray sticks). Software failed to model binding of SAM consistently with a rationale that these substitutions inactivate catalytic activity.

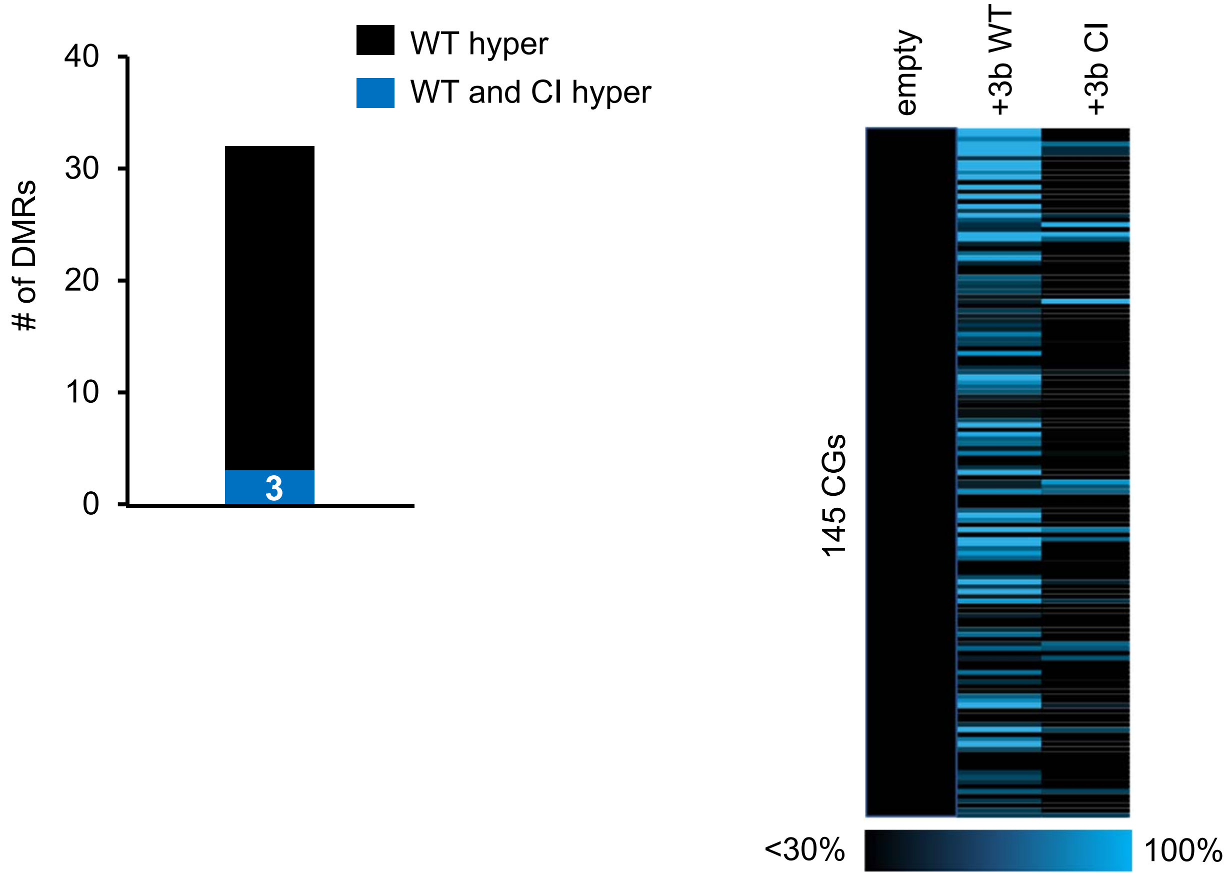

Sample	Paired-end reads before QC	Paired-end reads after QC and filtering	Aligned reads	Mapping efficiency	CpGs 10x	Average site coverage
DKO + 3b WT	22,124,090	21,503,310	19,940,974	92.73%	1,253,132	31.76
DKO + 3b CI	24,455,745	23,296,269	21,450,745	92.08%	1,329,699	34.48
DKO + empty vector	18,982,020	18,489,596	16,964,227	91.75%	1,169,971	30.59

Supplementary Fig. 2. Hypermethylated DMRs identified in Dnmt3a$^{-/-}$ Dnmt3b$^{-/-}$ lymphoma cells overexpressing *Dnmt3b*^{WT} and *Dnmt3b*^{CI}.
Left. The number of hypermethylated DMRs (≥3 consecutive DMCs in the same direction in the distance ≤100 bp, average DMC methylation change ≥30%, $p<0.05$) identified in Dnmt3a$^{-/-}$ Dnmt3b$^{-/-}$ (DKO) cell line overexpressing *Dnmt3b*^{WT} when compared to empty vector control. Overlapping DMRs between Dnmt3a$^{-/-}$ Dnmt3b$^{-/-}$ cell line overexpressing *Dnmt3b*^{WT} *and Dnmt3b*^{CI} are marked with blue. *Right*. Heatmap showing methylation difference of 145 CGs included in DMRs presented on left side. *Bottom*. Summary of data obtained from the sequenced Reduced Representation Bisulfite Sequencing libraries.

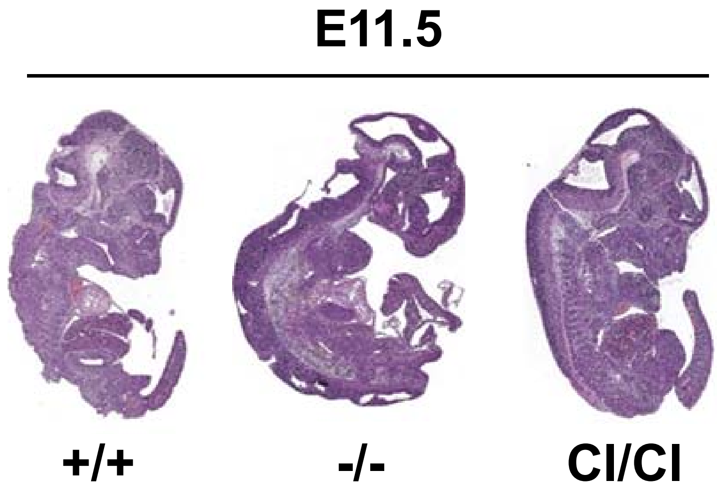

Supplementary Fig. 3 . Histological analysis of whole embryos at E11.5.
H&E stained sections prepared from *E11.5 Dnmt3b*$^{+/+}$, *Dnmt3b*$^{-/-}$ and *Dnmt3b*$^{CI/CI}$ whole embryos.

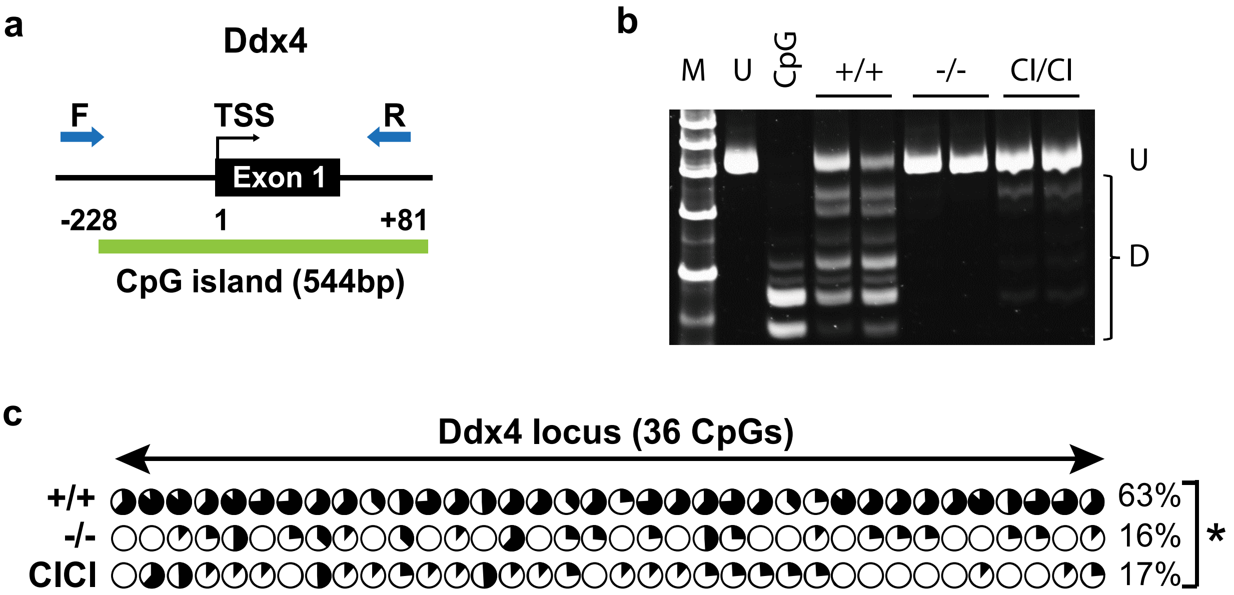

Supplementary Fig. 4. Methylation analysis of *Ddx4* locus using bisulfite sequencing.

a Graphical presentation depicting exon 1, transcription start site (TSS), CpG island and placement of F and R primers surrounding from -228 to +81 bp of *Ddx4* locus used for methylation analysis by bisulfite sequencing.

b COBRA of *Ddx4* in Dnmt3b$^{+/+}$ (+/+), Dnmt3b$^{-/-}$ (-/-) and Dnmt3b$^{CI/CI}$ (CI/CI) embryos and a fully methylated control (CpG). *Ddx4* was digested with *Bst*UI. Undigested (U) and digested (D) fragments correspond to unmethylated and methylated DNA, respectively.

c Bisulfite sequencing of PCR fragments generated using F and R primers *Ddx4* locus using DNA isolated from E11.5 *Dnmt3b*$^{+/+}$, *Dnmt3b*$^{-/-}$ and *Dnmt3b*$^{CI/CI}$ *embryos.* Each pie represents a CpG dinucleotide and each wedge of pie represents the sequence of an individual allele. Black wedges correspond to methylated CpG dinucleotides and white wedges represent unmethylated CpGs. Results derived from 8-10 clones are shown (*$p<0.05$).

a

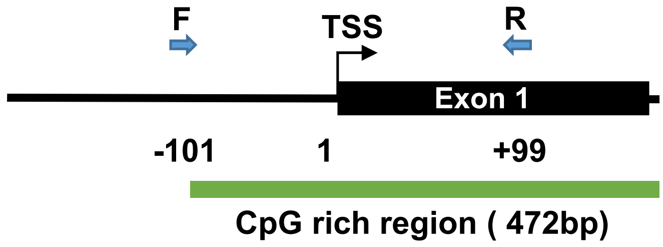

b

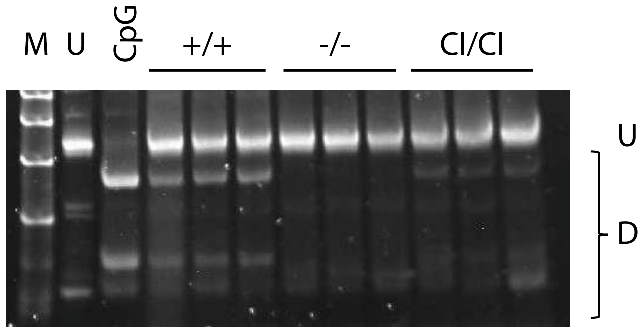

c

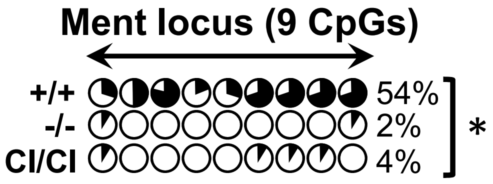

Supplementary Fig. 5. Methylation analysis of *Ment* locus using bisulfite sequencing.

a Graphical presentation depicting exon 1, transcription start site (TSS), CpG rich region and placement of F and R primers surrounding from -101 to +99 bp of *Ment* locus used for methylation analysis by bisulfite sequencing.

b COBRA of *Ment* in $Dnmt3b^{+/+}$ (+/+), $Dnmt3b^{-/-}$ (-/-) and $Dnmt3b^{CI/C}$I (CI/CI) embryos and a fully methylated control (CpG). *Ment* was digested with *Taq*I. Undigested (U) and digested (D) fragments correspond to unmethylated and methylated DNA, respectively.

c Bisulfite sequencing of PCR fragments generated using F and R primers *Ment* locus using DNA isolated from E11.5 *$Dnmt3b^{+/+}$*, *$Dnmt3b^{-/-}$* and *$Dnmt3b^{CI/CI}$ embryos.* Each pie represents a CpG dinucleotide and each wedge of pie represents the sequence of an individual allele. Black wedges correspond to methylated CpG dinucleotides and white wedges represent unmethylated CpGs. Results derived from 8-10 clones are shown (*$p<0.05$).

Sample	Paired-end reads before QC	Paired-end reads after QC and filtering	Aligned reads	Mapping efficiency	CpGs 15x	Avg site coverage
+/+ #1	31,362,284	27,699,640	25,694,963	92.76%	1,116,838	30.94
+/+ #2	34,304,787	30,127,858	28,028,359	93.03%	1,253,741	29.18
-/- #1	31,490,783	27,240,676	25,214,669	92.56%	1,196,041	28.21
-/- #2	25,018,398	21,690,529	20,226,933	93.25%	1,064,030	26.88
CI/CI #1	33,409,523	29,374,119	27,250,269	92.77%	1,231,404	30.07
CI/CI #2	40,375,417	35,606,665	33,235,669	93.34%	1,308,882	32.69

Supplementary Fig. 6. Summary of data obtained from the sequenced Reduced Representation Bisulfite Sequencing libraries.

Post sequencing quality check of RRBS data obtained from DNA of *Dnmt3b*$^{+/+}$, *Dnmt3b*$^{-/-}$ and *Dnmt3b*$^{CI/CI}$ E11.5 embryos (n=2 each). The last two columns indicate the number of CpGs sequenced at least 15x and average site coverage obtained from reads that mapped uniquely in the genome.

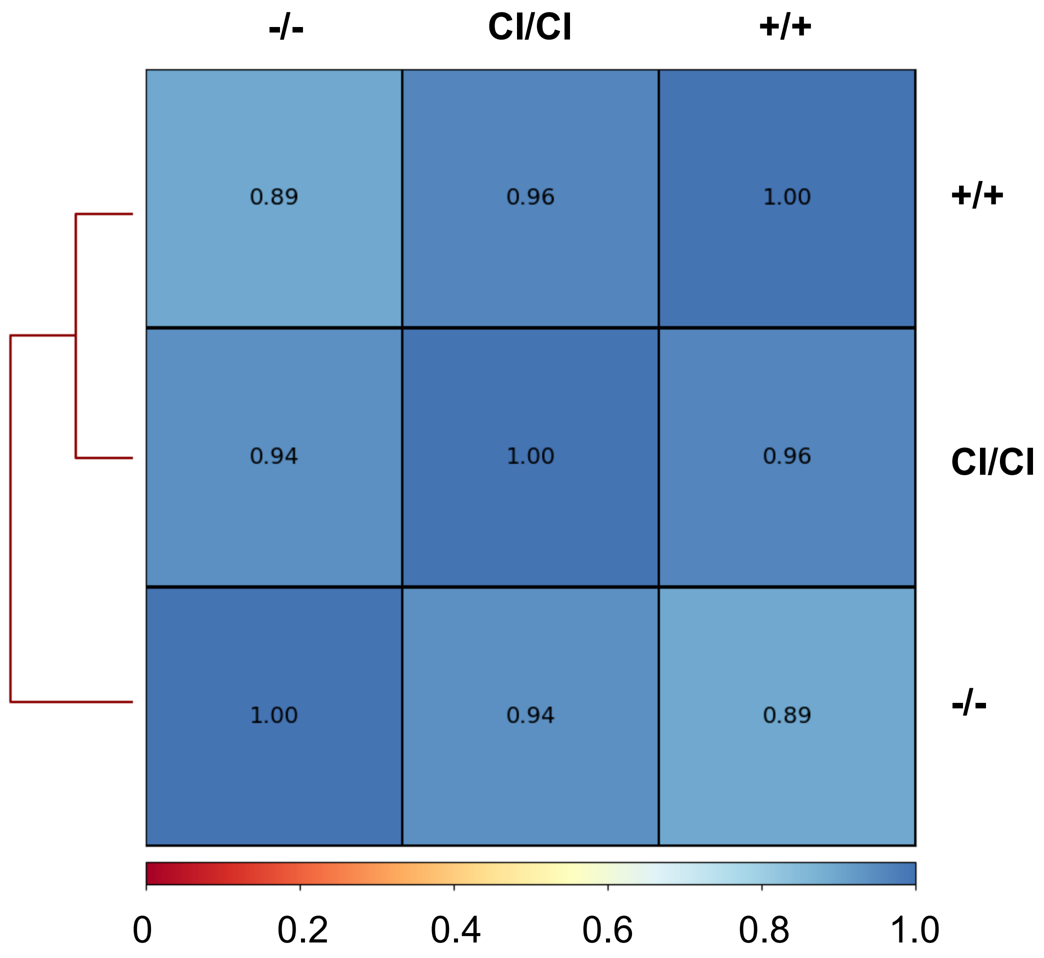

Supplementary Fig. 7. Pearson correlation coefficients and hierarchical clustering of genome-wide methylation data. Clustering of the samples and correlation analysis was based on methylation values of all CGs present in all analyzed samples (Dnmt3b$^{+/+}$ (+/+) embryos (n=2), Dnmt3b$^{CI/CI}$ (CI/CI) embryos (n=2) and Dnmt3b$^{-/-}$ (-/-) embryos (n=2) covered at least 15x (>8x10^{5}). Pearson correlation analysis was performed using deepTools packages multiBigWigsummary and plotCorrelation.

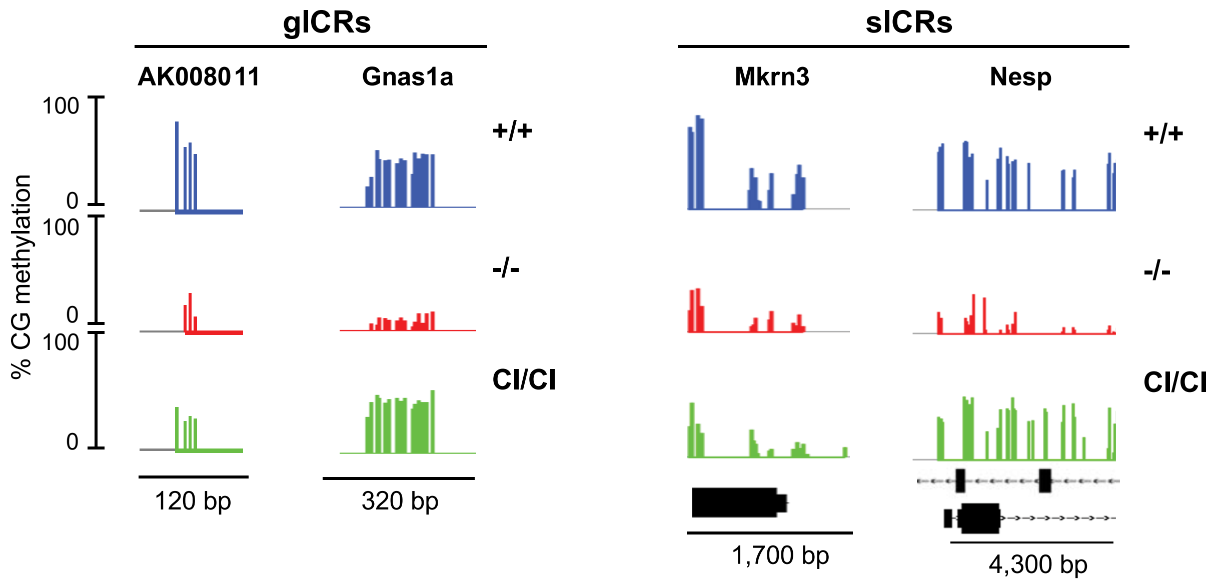

Supplementary Fig. 8. Methylation analysis of selected germline and somatic ICRs.

Percentage of CG methylation in gICRs (*AK008011* and *Gnas1a*) and sICRs (*Mkrn3* and *Nesp*) loci obtained by RRBS from genomic DNA isolated from E11.5 *Dnmt3b*$^{+/+}$, *Dnmt3b*$^{-/-}$ and *Dnmt3b*$^{CI/CI}$ *embryos*. Methylation levels were visualized by IGB software and are presented as percentage of methylation for indicated regions.

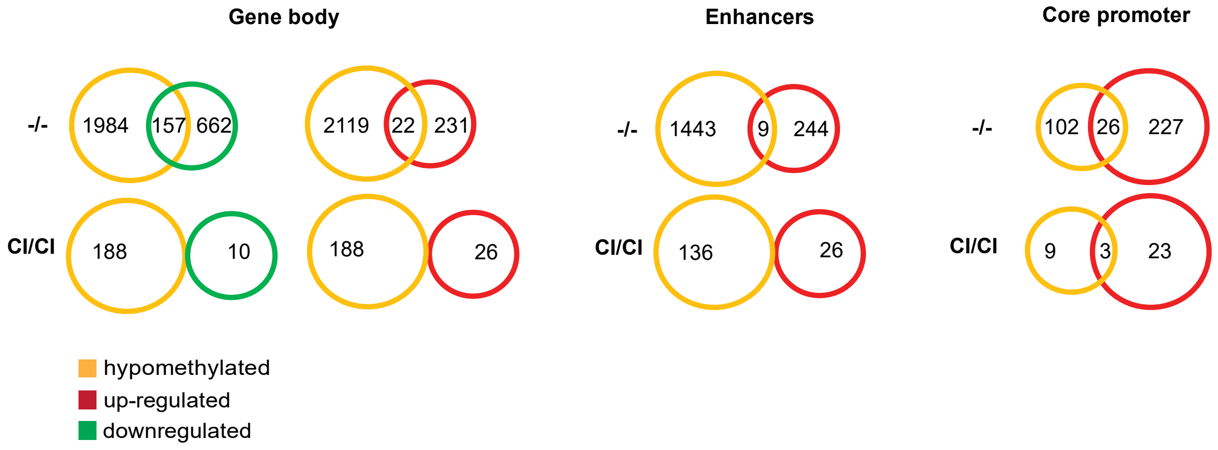

Supplementary Fig. 9. Overlap of methylation and gene expression changes in E11.5 embryos of different genotypes in various genomic elements.
Venn diagrams displaying a number of indicated genomic elements associated with DMRs and differential expression in *Dnmt3b*$^{-/-}$ and *Dnmt3b*$^{CI/CI}$ embryos relative to *Dnmt3b*$^{+/+}$ counterparts. Hypomethylated changes are indicated by yellow circles, decreased gene expression by green circles and increased expression by red circles.

Genomic Distribution of DMRs

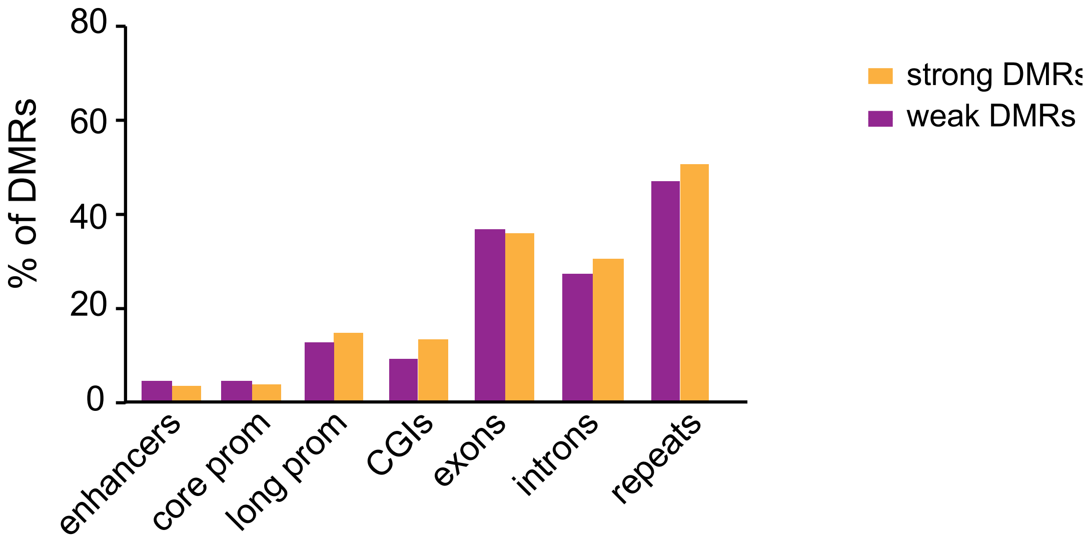

Supplementary Fig. 10. Percentage of DMRs distribution among indicated genomic element.

The number of DMRs (≥ 3 consecutive DMCs in the same direction in the distance ≤ 200 bp, average DMC methylation change ≥ 30%, $p<0.05$) identified in *Dnmt3b*$^{-/-}$ and *Dnmt3b*$^{CI/CI}$ E11.5 embryos relative to *Dnmt3b*$^{+/+}$ counterparts. Distribution of loci strongly ("*strong*") and weakly ("*weak*") dependent on catalytic activity of Dnmt3b is shown.

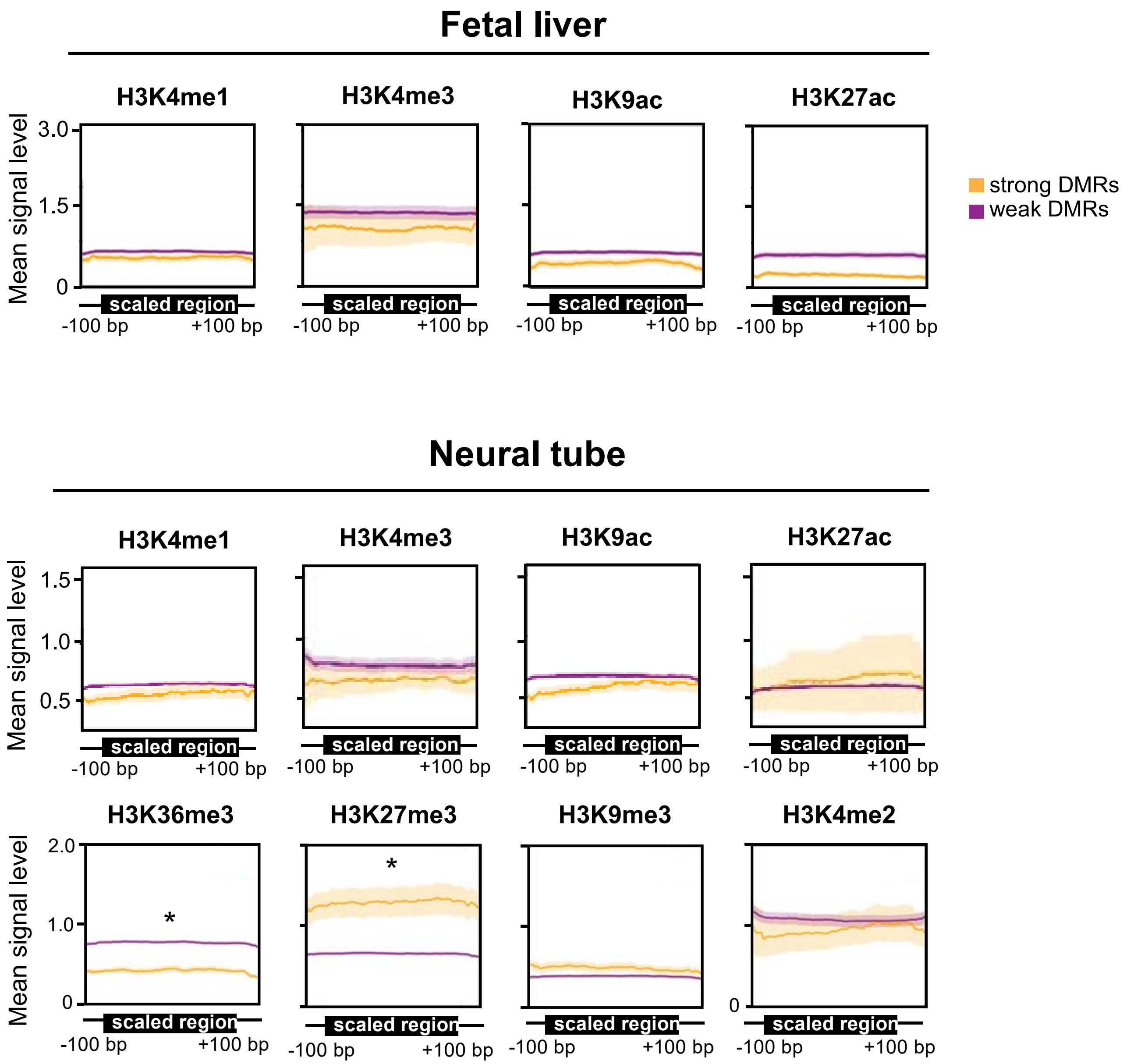

Supplementary Fig. 11. Enrichment of "*strong*" DMRs and "*weak*" DMRs for chromatin marks in E11.5 *Dnmt3b*$^{+/+}$ embryonic tissues.

Enrichment of "*strong*" DMRs and "*weak*" DMRs for indicated chromatin marks in fetal liver and neural tube of E11.5 *Dnmt3b*$^{+/+}$ embryos as detected by analysis of ChIP-seq data. Mean levels ± SEM of histone marks in indicated genomic areas is shown. Plots shows profiles for DMRs scaled to the same length and surrounding 100 bp unscaled regions. Statistical significance was calculated by Welch's t-test; significance of $p<0.05$ is depicted by *. SEM values are presented as shading around mean value line.

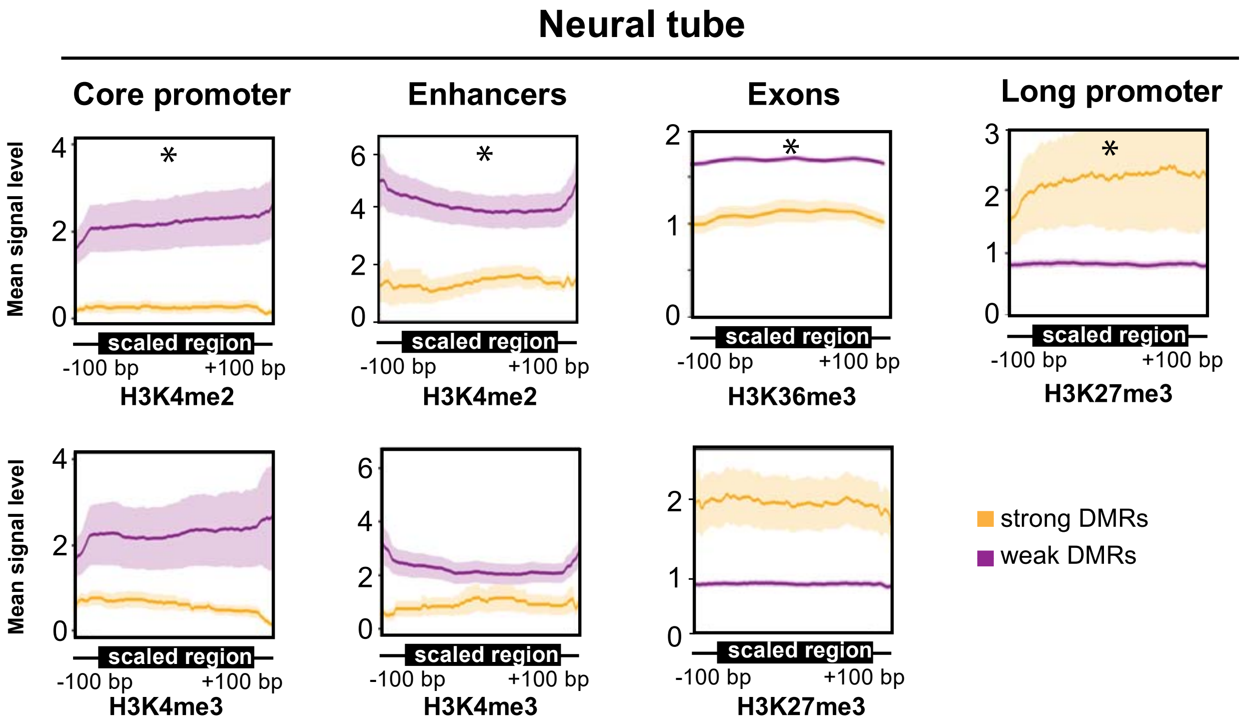

Supplementary Fig. 12. Enrichment of “*strong”* DMRs and “*weak*” DMRs for chromatin marks in neural tube of E11.5 *Dnmt3b*$^{+/+}$ embryo.

Enrichment of “*strong”* DMRs and “*weak*” DMRs for indicated chromatin marks within indicated genomic elements in neural tube of *Dnmt3b*$^{+/+}$ E11.5 embryos as detected by analysis of ChIP-seq data. Mean levels ± SEM of histone marks in indicated genomic areas is shown. Plots shows profiles for DMRs scaled to the same length and surrounding 100 bp unscaled regions. Statistical significance was calculated by Welch’s t-test; significance of $p<0.05$ is depicted by *. SEM values are presented as shading around mean value line.

a

	# of CpG sites in WT E11.5 which gained meth >=30%	# of CpG sites in -/- E11.5 which are hypo >=30%	%	# of CpG sites in CI/CI E11.5 which are hypo >=30%	%
**ALL**	40,842	10,690	26	2,462	6
**exon**	8,091	2,997	37	683	8
**intron**	16,042	3,685	23	812	5
**repeat**	27,503	6,581	24	1,486	5
**long promoter**	2,655	1,079	41	322	12
**core promoter**	771	334	43	102	13
**CGI**	866	615	71	229	26
**enhancer**	1,217	442	36	136	11

b

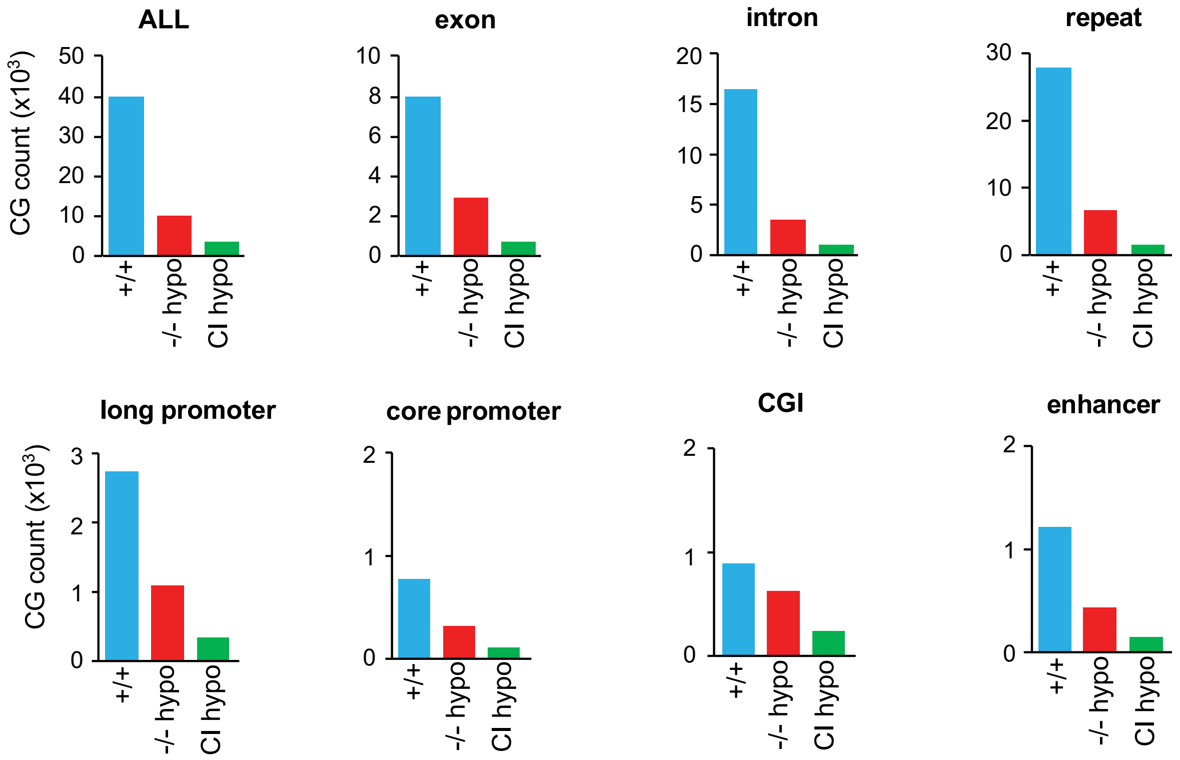

Supplementary Fig. 13. Methylation of CG dinucleotides and their distribution across various genomic elements in E11.5 embryos.

a Number of CG sites which gained at least 30% of methylation in WT at E11.5 when compared to E10.5 but not in Dnmt3b$^{-/-}$ or Dnmt3b$^{CI/CI}$ at E11.5. *De novo* methylation in CGIs is mostly Dnmt3b dependent (71% of all CG sites loose at least 30% methylation in Dnmt3b$^{-/-}$ embryos).

b Bar graphs showing number of *de novo* methylated events across indicated genomic elements dependent on Dnmt3b catalytic as well as accessory function of Dnmt3b.

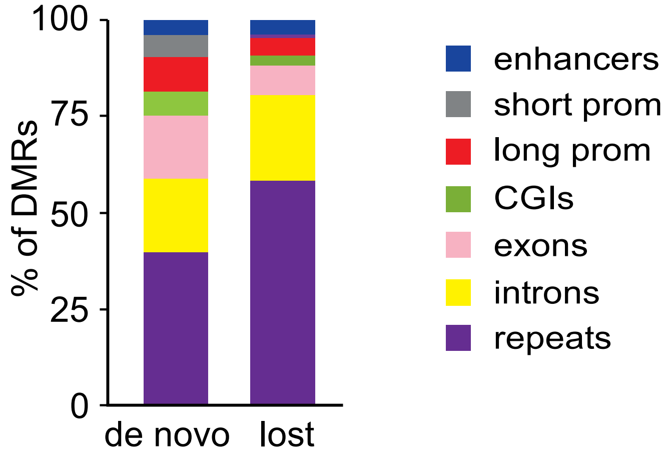

Supplementary Fig. 14. Genomic distribution of DMRs detected at E11.5 relative to E10.5 *Dnmt3b*$^{+/+}$ embryos.

Total number of DMRs with gains and losses in methylation were annotated to indicated genomic elements.

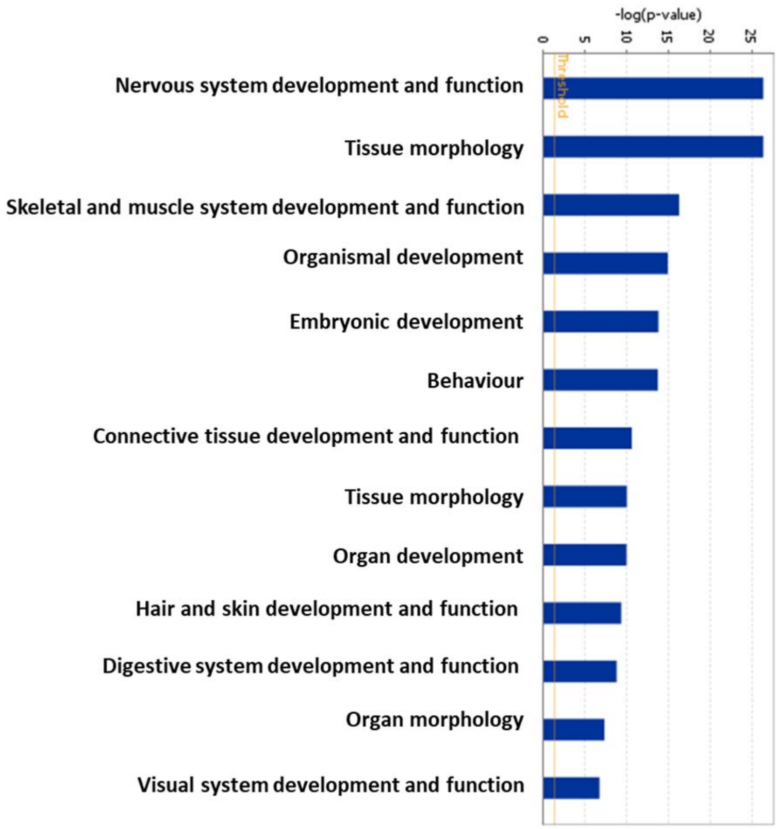

Suplementary Fig. 15: IPA analysis of downregulated genes in Dnmt3b$^{-/-}$ embryos at E11.5. Analysis was based on 819 genes downregulated in Dnmt3b$^{-/-}$ embryos compared to *Dnmt3b*$^{+/+}$ embryos, out of these, 771 genes were recognized by IPA.

Biological process	p-value	Gene list
reproductive process	5.00E-12	Rnf17, Fgf8, Hoxa11, Asz1, Tex19.1, Hmga2, Mov10l1, Hormad2, Hormad1, Wnt9b, Piwil2, Shh, Tex12, Lin28a
meiotic cell cycle	2.68E-11	Piwil2, Asz1, Tex19.1, Hmga2, Hormad1, Mov10l1, Hormad2
regulation of gene expression	1.00E-08	Fos, Fgf8, Hoxa11, Asz1, Tex19.1, Hmga2, Lin28b, Mov10l1, Tcf15, Piwil2, Shh, Sost, Cdkn1a, Smtnl1, Hist1h1b, Sp6, Lin28a
gene silensing by RNA	2.22E-08	Piwil2, Asz1, Lin28b, Mov10l1, Lin28a
canonical Wnt signalin pathway	1.43E-06	Frzb, Shh, Fgf8, Wnt9b
piRNA metabolic process	4.79E-06	Piwil2, Asz1, Mov10l1
negative regulat on of macromolecule metabolic process	1.26E-04	Piwil2, Shh, Cdkn1a, Asz1, Smtnl1, Hist1h1b, Hmga2, Lin28b, Mov10l1, Lin28a
negative regulat on of developmental process	1.34E-04	Frzb, Shh, Fgf8, Cdkn1a, Hmga2, Lin28a, Wnt9b
non-canonical Wnt signaling pathway	8.42E-04	Frzb, Wnt9b

Suplementary Fig. 16. IPA and Gene ontology analysis of genes up-regulated specifically in E11.5 *Dnmt3b*$^{-/-}$.

IPA analysis of genes specifically upregulated in E11.5 *Dnmt3b*$^{-/-}$ (FC≥2, $p<0.05$) but not in *Dnmt3b*$^{CI/CI}$ relative to *Dnmt3b*$^{+/+}$ embryos. Genes in subcategory “Embryonic development” were further analyzed by gene ontology. Main categories with significance and gene identities is shown.

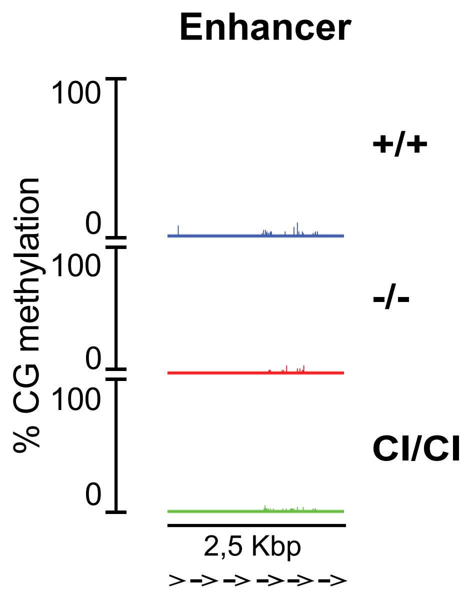

Supplementary Fig. 17. DNA methylation analysis of putative enhancer of Wnt9b.
DNA methylation scores obtained for 2.5 kbp locus harboring *Wnt9b* gene enhancer from RRBS analysis of E11.5 *Dnmt3b*$^{+/+}$, *Dnmt3b*$^{-/-}$ and *Dnmt3b*$^{CI/CI}$ embryos. Data were visualized by IGB software and are presented as percentage of methylation for indicated regions. No significant methylation changes were observed between genetic settings.

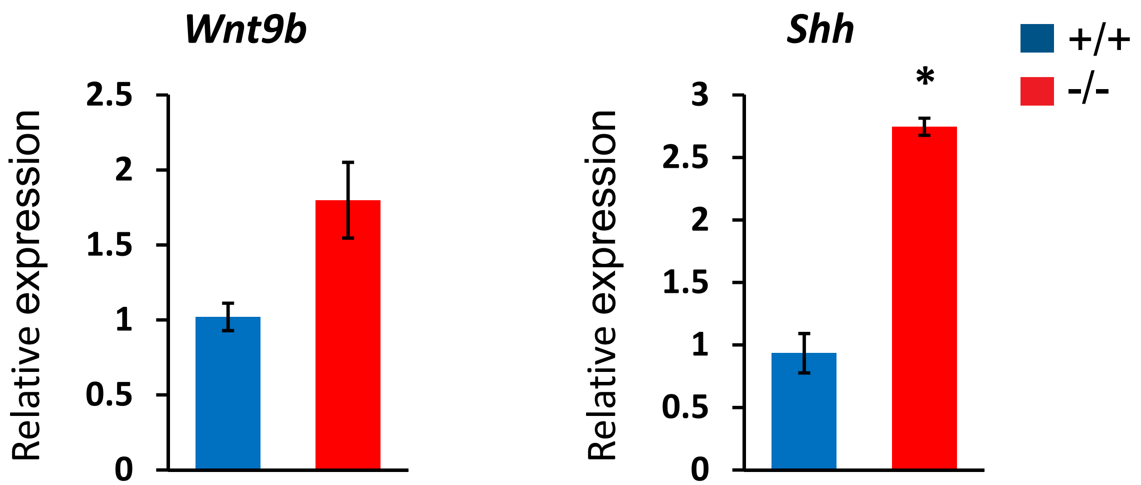

Supplementary Fig. 18. Expression of Shh and Wnt9b in *Dnmt3b*$^{-/-}$ embryos at E10.5.

Wnt9b and Shh expression analyzed by qRT-PCR in Dnmt3b$^{+/+}$ (n=4) and Dnmt3b$^{-/-}$ (n = 2) E10.5 embryos, normalized to β-actin and presented as means ± SEM (*p<0.001).

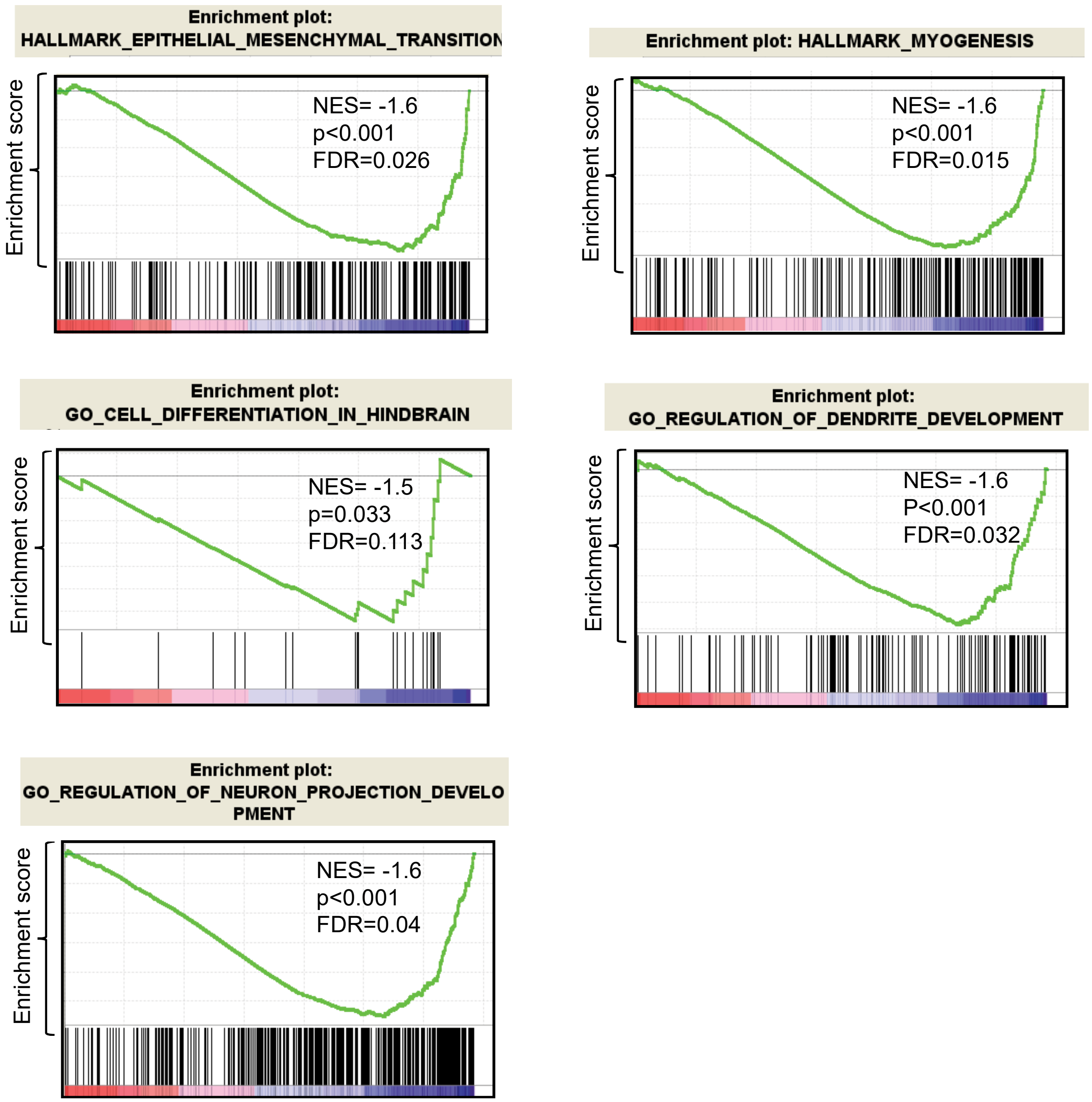

Supplementary Fig. 19. Developmental pathways in Dnmt3b$^{-/-}$ embryos are downregulated.

GSEA plots of gene sets specific to developmental pathways which gets downregulated in *Dnmt3b*$^{-/-}$ compared to *Dnmt3b*$^{+/+}$ embryos (E11.5).

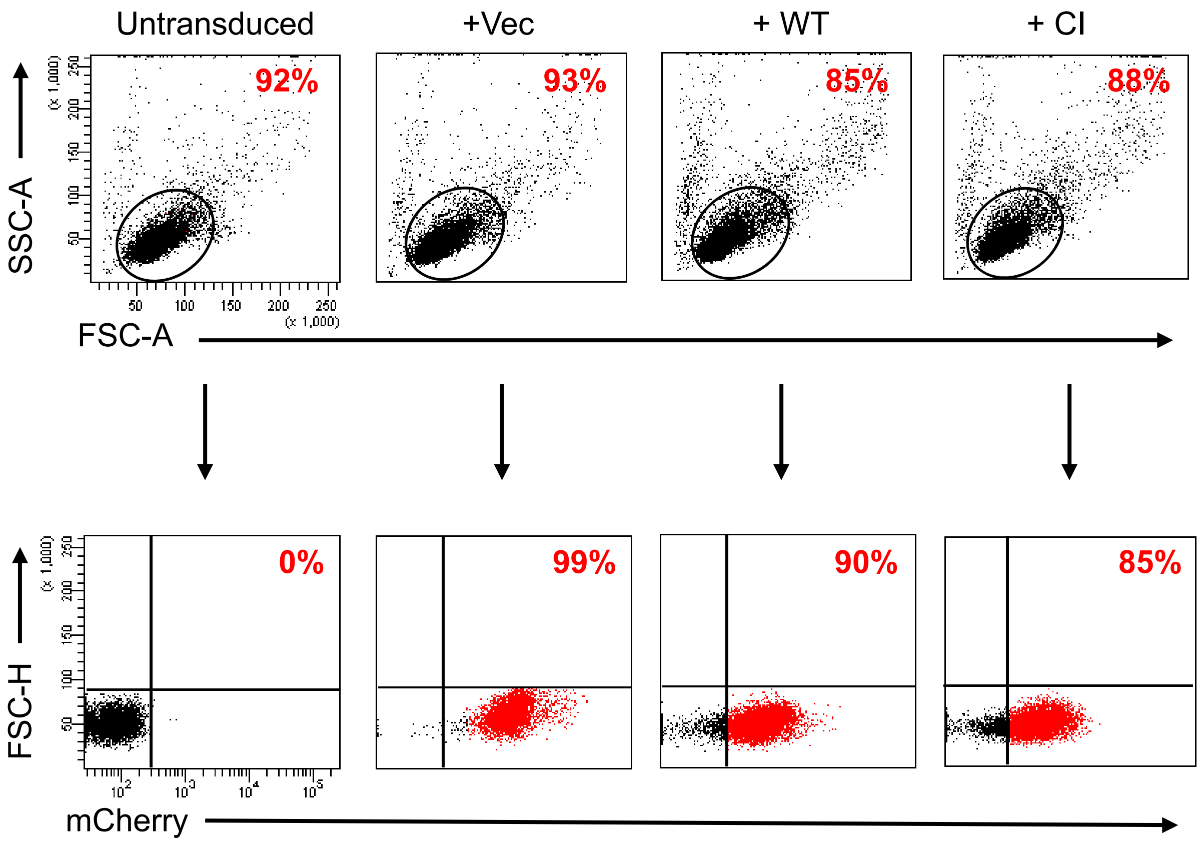

Supplementary Fig. 20. Gating strategy for the flow cytometric analysis of transduction efficiency in T cell lymphoma lines. Mouse T cell lymphoma cells from *Dnmt3b*$^{-/-}$ were transduced with lentiviral vectors carrying sequence for wild-type Dnmt3b (WT), Dnmt3b P705V, C706D (CI) and vector control (Vec). Efficiency of transduction was measured as mCherry signal. Percentages of mCherry positive cells are shown in the top right quadrant in red. FSC, forward light scatter; SSC, side light scatter. Untranduced cells serve as a negative control.
