# Supplementary material for: Catalytically inactive Dnmt3b rescues mouse embryonic development by accessory and repressive functions
Source: Nat Commun. 2019 Sep 26;10:4374. doi: 10.1038/s41467-019-12355-7 (PMC6763448; doi:10.1038/s41467-019-12355-7)
Supplement: Supplementary file 2 — Reporting Summary [file 41467_2019_12355_MOESM2_ESM.pdf]

nature research

Corresponding author(s): Rene Opavsky

Last updated by author(s): Sep 3, 2019

Reporting Summary

Nature Research wishes to improve the reproducibility of the work that we publish. This form provides structure for consistency and transparency in reporting. For further information on Nature Research policies, see Authors & Referees and the Editorial Policy Checklist.

Statistics

For all statistical analyses, confirm that the following items are present in the figure legend, table legend, main text, or Methods section.

n/a	Confirmed	
☐	☒	The exact sample size (*n*) for each experimental group/condition, given as a discrete number and unit of measurement
☐	☒	A statement on whether measurements were taken from distinct samples or whether the same sample was measured repeatedly
☐	☒	The statistical test(s) used AND whether they are one- or two-sided<br>*Only common tests should be described solely by name; describe more complex techniques in the Methods section.*
☐	☒	A description of all covariates tested
☒	☐	A description of any assumptions or corrections, such as tests of normality and adjustment for multiple comparisons
☐	☒	A full description of the statistical parameters including central tendency (e.g. means) or other basic estimates (e.g. regression coefficient) AND variation (e.g. standard deviation) or associated estimates of uncertainty (e.g. confidence intervals)
☐	☒	For null hypothesis testing, the test statistic (e.g. *F*, *t*, *r*) with confidence intervals, effect sizes, degrees of freedom and *P* value noted<br>*Give P values as exact values whenever suitable.*
☒	☐	For Bayesian analysis, information on the choice of priors and Markov chain Monte Carlo settings
☒	☐	For hierarchical and complex designs, identification of the appropriate level for tests and full reporting of outcomes
☐	☒	Estimates of effect sizes (e.g. Cohen's *d*, Pearson's *r*), indicating how they were calculated

Our web collection on statistics for biologists contains articles on many of the points above.

Software and code

Policy information about availability of computer code

Data collection: *Provide a description of all commercial, open source and custom code used to collect the data in this study, specifying the version used OR state that no software was used.*

Data analysis: *Provide a description of all commercial, open source and custom code used to analyse the data in this study, specifying the version used OR state that no software was used.*

For manuscripts utilizing custom algorithms or software that are central to the research but not yet described in published literature, software must be made available to editors/reviewers. We strongly encourage code deposition in a community repository (e.g. GitHub). See the Nature Research guidelines for submitting code & software for further information.

Data

Policy information about availability of data

All manuscripts must include a data availability statement. This statement should provide the following information, where applicable:

- Accession codes, unique identifiers, or web links for publicly available datasets
- A list of figures that have associated raw data
- A description of any restrictions on data availability

The RRBS and RNA-seq data are deposited at the NCBI Gene Expression Omnibus database under the number GSE132952.

Field-specific reporting

Please select the one below that is the best fit for your research. If you are not sure, read the appropriate sections before making your selection.

☒ Life sciences ☐ Behavioural & social sciences ☐ Ecological, evolutionary & environmental sciences

October 2018

Life sciences study design

All studies must disclose on these points even when the disclosure is negative.

Sample size	Mouse crosses were performed to obtain 436 mice. Data were analyzed using Student's t test. Data were considered statistically significant when p<0.05.
Data exclusions	No data were excluded.
Replication	Generation of knockin allele was performed independently in BL6 mouse strain provideng the same results.
Randomization	mouse were selected based on genotypes. All crosses were het x het.
Blinding	Investigators were double blinded. Mouse tailing was performed by different investigators than genotyping.

Reporting for specific materials, systems and methods

We require information from authors about some types of materials, experimental systems and methods used in many studies. Here, indicate whether each material, system or method listed is relevant to your study. If you are not sure if a list item applies to your research, read the appropriate section before selecting a response.

Materials & experimental systems

n/a	Involved in the study	
☐	☒	Antibodies
☐	☒	Eukaryotic cell lines
☒	☐	Palaeontology
☐	☒	Animals and other organisms
☒	☐	Human research participants
☒	☐	Clinical data

Methods

n/a	Involved in the study	
☒	☐	ChIP-seq
☐	☒	Flow cytometry
☒	☐	MRI-based neuroimaging

Antibodies

Antibodies used	Dnmt1 (ab188453, Abcam), Dnmt3a (SC-20703, Santa Cruz), Dnmt3b (PA1-884, Thermo Fisher), Dnmt3L (SC-393603, Santa Cruz), Fgf8 (MAB323, R&D Systems), Wnt9b (AF3669, R&D Systems), Hsc70 (SC-7298, Santa Cruz), anti-FLAG antibody (F3165, Sigma).
Validation	antibodies were validated by dot plot and using recombined proteins as a control

Eukaryotic cell lines

Policy information about cell lines

Cell line source(s)	Mouse Dnmt3b-/- and Dnmt3a-/-;Dnmt3b-/- lymphoma cell lines were generated in the lab. 293T cell line was purchased from Invitrogen/Takara.
Authentication	Cell line was periodically checked for karyotype. Only early passages were used.
Mycoplasma contamination	Cell lines were periodically tested for mycoplasma contamination and were found to be negative at all times.
Commonly misidentified lines (See ICLAC register)	none

Animals and other organisms

Policy information about studies involving animals; ARRIVE guidelines recommended for reporting animal research

Laboratory animals	Mouse FVB and BL6 strains were used.
Wild animals	*Provide details on animals observed in or captured in the field; report species, sex and age where possible. Describe how animals were caught and transported and what happened to captive animals after the study (if killed, explain why and describe method; if released, say where and when) OR state that the study did not involve wild animals.*

Field-collected samples	*For laboratory work with field-collected samples, describe all relevant parameters such as housing, maintenance, temperature, photoperiod and end-of-experiment protocol OR state that the study did not involve samples collected from the field.*
Ethics oversight	The study was approved by Institutional Animal Care and Use Committee (IACUC), University of Florida, Gainesville

Note that full information on the approval of the study protocol must also be provided in the manuscript.

Flow Cytometry

Plots

Confirm that:

☒ The axis labels state the marker and fluorochrome used (e.g. CD4-FITC).

☒ The axis scales are clearly visible. Include numbers along axes only for bottom left plot of group (a 'group' is an analysis of identical markers).

☐ All plots are contour plots with outliers or pseudocolor plots.

☒ A numerical value for number of cells or percentage (with statistics) is provided.

Methodology

Sample preparation	Mouse T cell lymphoma cells were established from Dnmt3b-/- mice. Cells were transduced with lentiviral vectors carrying sequence for wild-type Dnmt3b, Dnmt3b P705V, C706D and vector control.
Instrument	BD LSR Fortessa, model 647794L5 (ICBR Bioinformatics Core (UF, Florida))
Software	BD FACS Diva (ICBR Bioinformatics Core (UF, Florida))
Cell population abundance	NA
Gating strategy	Cells were gated for lymphocytes (SSC-A vs FSC-A). Boundaries between mCherry positive and negative cells were setup based on untransduced control.

☒ Tick this box to confirm that a figure exemplifying the gating strategy is provided in the Supplementary Information.
