# Supplementary material for: Catalytically inactive Dnmt3b rescues mouse embryonic development by accessory and repressive functions
Source: Nat Commun. 2019 Sep 26;10:4374. doi: 10.1038/s41467-019-12355-7 (PMC6763448; doi:10.1038/s41467-019-12355-7)
Supplement: Supplementary file 4 — Description of Additional Supplementary Files [file 41467_2019_12355_MOESM4_ESM.pdf]

Description of Additional Supplementary Files

File Name: Supplementary Data 1

Description: Results of CG methylation analysis underlying heat map in Figure 2g, determined by RRBS in Dnmt3b-/-, Dnmt3bCI/CI and Dnmt3bWT/WT E11.5 embryos.

File Name: Supplementary Data 2

Description: Genomic coordinates and methylation levels of mouse gICRs and sICRs detected in Dnmt3b-/-and Dnmt3bCI/CI E11.5 embryos.

File Name: Supplementary Data 3

Description: Log2(FPKM) values of genes underlying heat map and hierarchical clustering in Figure 3b determined by RNA-seq in Dnmt3b-/-, Dnmt3bCI/CI and Dnmt3bWT/WT E11.5 embryos.

File Name: Supplementary Data 4

Description: Results of RNA-seq analysis underlying heat maps in Figure 3e. File contains FPKM values detected in Dnmt3bWT/WT E11.5 embryos and Fold change expression values of Dnmt3b-/- E11.5 embryos normalized Dnmt3bWT/WT embryos.

File Name: Supplementary Data 5

Description: Genomic position and methylation levels of DMRs detected in Dnmt3b-/-and Dnmt3bCI/CI E11.5 embryos.

File Name: Supplementary Data 6

Description: Sequences of primers used for Real-time qRT-PCR, Bisulfite sequencing/COBRA and genotyping.
