# Supplementary material for: Catalytically inactive Dnmt3b rescues mouse embryonic development by accessory and repressive functions
Source: Nat Commun. 2019 Sep 26;10:4374. doi: 10.1038/s41467-019-12355-7 (PMC6763448; doi:10.1038/s41467-019-12355-7)
Supplement: Supplementary file 3 — Peer Review File [file 41467_2019_12355_MOESM3_ESM.pdf]

Reviewers' comments:

Reviewer #1 (Remarks to the Author):

In this study Opavsky and colleagues investigate the noncatalytic functions of Dnmt3b using a catalytic deficient mouse model. They find that the catalytic functions of Dnmt3b are dispensable for development as mice lacking this activity are viable in contrast to complete knockout mice (which die at E11.5). They further report that the noncatalytic functions of Dnmt3b include an accessory role (which may facilitate methylation through other Dnmts), as well as a repressor role (which prevents aberrant activation of wnt and shh pathways). The study is interesting and the first to investigate the noncatalytic functions of Dnmt3b during mouse development. The study will benefit from further characterization of embryos and validation of some targets in vivo, as well as proposing clearer models for the accessory and repressor functions of Dnmt3b. I think with some revisions, it will be suitable for publication in Nature Communications.

Major points:

1- Mouse model: How well is it established that the two aa substitutions render Dnmt3b catalytically inactive? Can the authors provide some in vitro or in vivo tests, using Dnmt3b CI in in vitro methylation assay, or overexpressing Dnmt3b CI in Dnmt TKO ESCs and test for methylation levels? This will help them make a better case for the “accessory role” of 3b later in the paper.

2- Analysis of mouse model: What is the specific organ/organs affected in KO mice? Authors should comment more on how their models influence the development of this organ/organs. Also, CI are smaller in size, this should be appreciated and commented on as it relates to significance of catalytic roles of 3b. Do later in life these mice catch up in size, how long do they live?

3- Analysis of mouse model: Are the CI mice fertile? Dnmt3L KO mice are infertile. If a role equivalent to Dnmt3L is proposed for Dnmt3b, then it would be useful to provide some data on the fertility of CI mice.

4- Molecular work: Most conclusions in the paper are made from global analysis of RNA and DNA from the whole embryo (not specific organs). At E11.5 the KO and CI embryos have very different developmental appearance so it is expected to find different gene expression and epigenetic marks. Wouldn't the study have benefited if the authors had looked at expression at early stages of phenotype manifestation, where embryos look normal but KO are destined to die and CI destined to develop. Authors should comment more on this?

5- Molecular work: In figure 4 and 5 why different data sets for histone marks are used. Why going from whole embryo histone marks, to neural tube marks and then to fetal liver. Why not stick with one set of data (whole embryo), especially when comparing gene expression and methylation changes from whole embryo?

6- Molecular work: There are 253 up and 819 down regulated genes? Focus is given on the up regulated genes, and the 819 down regulated genes are not elaborated upon? I don't see a good rationale for this. The 819 genes could be critical too.

7- Molecular work: Wnt9b, Fgf8 and Shh are reported as upregulated in KO embryos and are validated by qPCR and western blot on total embryo samples. Can some IF or in situ be performed to see where in the embryo these genes are upregulated? If lethality is due to these two pathways, are they aberrantly expressed in the organs affected in KO embryos at E11.5?

8- Proposed models: The accessory role of dnmt3b should be better defined (ideally show that dnmt3a occupies sites that would normally be occupied by 3b, or show that there is a recruiting function for 3b). What is the accessory role? I understand this may be extensive work for publication of this study at Nat. Comm. , but authors at least can more clearly comment on this in the revised manuscript.

9- Proposed models: The repressor function should be better defined. How 3b promotes repression non-catalytically? Does it increase the level of H3K27me3 or other repressive marks on the upregulated genes? Does it affect the recruitment of any histone modifying enzymes/complex or affect chromatin accessibility? What is the repressor role? I understand this may be extensive work for publication of this study at Nat. Comm., but authors can more clearly comment on this in the discussion of the revised manuscript, it is very fuzzy to me now.

Minor points:

1- Global measurement of 5mC levels in WT, CI and KO embryos by dot blot or other methods would be useful to show the effect of Dnmt3b loss on global 5mC level.

2- In panels 7i and 7j (where WT or CI 3b are overexpressed in KO cells) the legend in the panel should be properly labeled to show that empty vector control is used.

Anonymous (not signed)

Reviewer #2 (Remarks to the Author):

The submitted manuscript by Nowialis and Lopusna et al details a methylation independent function of Dnmt3b using mouse models. Specifically, substitution of wild-type Dnmt3b with a catalytically-inactive version allows mice to survive embryonic development. Moreover, catalytically-inactive Dnmt3b rescues a significant portion of DNA hypomethylation observed in Dnmt3b-null mice, suggesting that Dnmt3b influences methylation patterns independent of catalytic activity. They ultimately link this alternate Dnmt3b function to two genes involved in embryonic development, Fgf8 and Wnt3b. Altogether, the authors appear to have carried out experiments meticulously, leaving this reviewer with only one major comment and a few minor ones.

Major comment:

While the authors nicely demonstrate linkage of wt/CI Dnmt3b expression with Wnt9b/Fgf8, at least one of these interactions should be confirmed. Binding of endogenous or overexpressed/tagged Dnmt3b ChIP should be performed if the authors wish to make the claim that Dnmt3b CI directly regulates Fgf8/Wnt9b, especially due to the previously reported role of Fgf8/Wnt9b in embryonic lethality. This could be even further supported with confirmation of methylation at this locus.

Minor comments:

Scale in Fig 2D for chrom length (also misspelled)

Fig 2G may be made more convincing by supplementing with genome-wide correlation from all ~800k CpGs to show that CI correlates/clusters better with +/+ at a genome-wide level.

Weighted Venns would be helpful in Fig 3A/C to emphasize the fewer number of changes in CI/CI relative to -/-.

It would be helpful in graphs showing the distribution of changes in genomic features such as enhancers and exons (e.g. Supplemental Fig.7/11) to be adjusted/normalized for their coverage by RRBS. This would make conclusions linked to enrichment at particular regions clearer.

This manuscript would benefit from general grammar check/proofing.

RESPONSE TO REVIEWERS

We would like to thank reviewers for their kind remarks regarding the importance of this work toward understanding the role of Dnmt3b catalytic activity in embryogenesis. We are also very thankful for the comments that helped us to make the revised manuscript much stronger. Major new findings included into the revised version of the manuscript are as follows:

- Results from global methylation analysis by RRBS of overexpression of Dnmt3b^{wt} and Dnmt3b^{CI} in *Dnmt3a*$^{-/-}$*; Dnmt3b*$^{-/-}$ lymphoma cells (**Supplementary Fig. 2**)
- Data on weights of *Dnmt3b*$^{+/+}$ and *Dnmt3b*$^{CI/CI}$ mice at different stage of postnatal development (**Fig. 1e**).
- LC-MS/MS-MRM analysis of global 5mC levels *Dnmt3b*$^{+/+}$*, Dnmt3b*$^{-/-}$ and *Dnmt3b*$^{CI/CI}$ embryos (**Fig. 1j**).
- Analysis of locus-specific methylation of *Ddx4* and *Ment* promoters by COBRA and bisulfite sequencing (**Supplementary Figure 4, 5**).
- IPA analysis of genes down-regulated in *Dnmt3b*$^{-/-}$ embryos (**Supplementary Figure 15**).
- Results of ChIP analysis of Dnmt3b recruitment to *Wnt9b* promoter (**Fig. 7j**)
- Real-time RT-qPCR data of *Wnt9b* and *Shh* expression in embryos in E10.5 in **Supplementary fig. 18.**
- Weighted Venn diagrams in **Fig. 3a**, **Fig. 3c** and **Supplementary Fig. 9.**
- Pearson correlation and clustering of E11.5 embryos based on genome-wide methylation data (**Supplementary Fig. 7**).

The text underwent a substantial revision. All textual changes are written in blue.

Please find below our point-by-point response (regular text) to the reviewers' comments (bolded text).

Reviewer 1

Major points:

1- Mouse model: How well is it established that the two aa substitutions render Dnmt3b catalytically inactive? Can the authors provide some in vitro or in vivo tests, using Dnmt3b CI in in vitro methylation assay, or overexpressing Dnmt3b CI in Dnmt TKO ESCs and test for methylation levels? This will help them make a better case for the "accessory role" of 3b later in the paper.

It is important to note that amino acid (aa) substitution of the C657 in the coding sequence of mouse Dnmt3b has been used previously to inactivate its catalytic activity (CA) [**1**]. To eliminate any residual CA, we designed double, rather than single, aa substitution Dnmt3b$^{P656V/C657D}$ to prevent binding of SAM and disable methylation reaction. Such design was modelled after analogous double aa substitution used to inactivate catalytic activity of Dnmt3a in corresponding active site (Dnmt3a$^{P705V/C706D}$) [**2,**

3]. We next paid a particular attention to perform a careful *in silico* analysis, which suggested loss of catalytic activity for Dnmt3b$^{P656V/C657D}$ as described in the manuscript.

However, we agree with the reviewer that this is an important point that should be addressed functionally *in vitro* and *in vivo*. Thus, we utilized several approaches to address this point:

1. Because TKO ESCs proposed by reviewer are not available to us, we utilized *Dnmt3a*$^{-/-}$*; Dnmt3b*$^{-/-}$ double knockout cells (DKO) derived from mouse lymphomas instead to overexpress Dnmt3b *wild-type* (Dnmt3b^{WT}) and catalytically inactive (Dnmt3b^{CI}) and measure DNA methylation changes by RRBS. This analysis revealed that Dnmt3b^{CI} induced less than 10% of DMRs than WT when overexpressed *in vitro*. Some detected activity may be due to its accessory function, e.g. ability to recruit Dnmt1 and induce methylation. We presented this data in new **Supplementary Figure 2.**

2. To analyze Dnmt3b^{CI} in more physiologically relevant setting, we measured locus-specific methylation of two well-known target genes – *Ddx4* and *Ment* [**4, 5**] by COBRA and bisulfite sequencing on samples derived from embryos at E11.5. While both loci were heavily methylated in WT setting, we observed a severe reduction in DNA methylation in *Dnmt3b*$^{-/-}$ embryos. Importantly, methylation levels in *Dnmt3b*$^{CI/CI}$ embryos were indistinguishable from *Dnmt3b*$^{-/-}$ and severely reduced relative to WT embryos. This analysis strongly supports the idea that Dnmt3b^{CI} is catalytically inactive *in vivo*.

3. To further address effects of Dnmt3b^{CI} *in vivo*, we analyzed global levels of DNA methylation in embryos using LC-MS/MS-MRM analysis. This analysis showed reductions in global methyl-cytosine levels similar to *Dnmt3b*$^{-/-}$ embryos further supporting the idea that Dnmt3b^{CI} is catalytically inactive. We present this data in **Fig. 1j.**

Despite all these data, we cannot rule out the possibility that Dnmt3b^{CI} has a residual catalytic activity *in vivo*, which is very difficult to discern in particular due to its extensive role as accessory protein for other Dnmts at least in embryogenesis. We therefore took a note of this in the result section **(page 5).**

2- Analysis of mouse model: What is the specific organ/organs affected in KO mice? Authors should comment more on how their models influence the development of this organ/organs. Also, CI are smaller in size, this should be appreciated and commented on as it relates to significance of catalytic roles of 3b. Do later in life these mice catch up in size, how long do they live?

In the first submission, we presented histological data on defects observed in fetal heart (**Fig. 1h, Supplementary Fig. 3** of the revised manuscript) and molecular data in brain (**Fig. 7d** of the revised manuscript) of *Dnmt3b*$^{-/-}$ embryos. At the present time, we are unable to pinpoint a single organ solely responsible for the observed lethal phenotype –

a daunting task in hard to manipulate *in vivo* setting of mouse embryogenesis. Because we detected deregulation of major developmental pathways, such as *Wnt* and *hedgehog* signaling on the level of whole embryo, it is likely that several developing organs are affected which is e.g. manifested by observation of hemorrhage as presented in **Fig. 1h**. It is important to note that Wnt signaling is involved in all aspects of embryogenesis and ectopic overexpression of Wnt9b leads to embryonic death at E11–E12.5 primarily due to cardiac insufficiency and hemorrhage [**6**].

Cell-type specific knockout of Dnmt3b or other complex approaches could further address this point in the future.

In the revised version, we added requested new data showing that $Dnmt3b^{CI/CI}$ mice remain smaller with ~20% in the weight reduction and such difference remains similar over the lifetime (**Fig. 1e**). Because the focus of this manuscript is the role of Dnmt3b in prenatal development and we feel that the manuscript is already “data heavy”, we do not elaborate on this observation further.

3- Analysis of mouse model: Are the CI mice fertile? Dnmt3L KO mice are infertile. If a role equivalent to Dnmt3L is proposed for Dnmt3b, then it would be useful to provide some data on the fertility of CI mice.

Both male and females $Dnmt3b^{CI/CI}$ mice are fertile and have normal lifespan. We added a comment into the result section (**page 6**).

4- Molecular work: Most conclusions in the paper are made from global analysis of RNA and DNA from the whole embryo (not specific organs). At E11.5 the KO and CI embryos have very different developmental appearance so it is expected to find different gene expression and epigenetic marks. Wouldn’t the study have benefited if the authors had looked at expression at early stages of phenotype manifestation, where embryos look normal but KO are destined to die and CI destined to develop. Authors should comment more on this?

We agree with the reviewer that molecular events should be assessed before major manifestations of phenotypes at which point too many events may be secondary to original molecular change. We chose E11.5 for molecular analysis because at this point a majority of $Dnmt3b^{-/-}$ are viable with lethal phenotype appearing mainly at E12.5 and later. All embryos used for molecular analysis were viable as determined by heartbeat and tissue coloration. Nonetheless, based on reviewer’s recommendation, we now present molecular analysis of Wnt9b and Shh at E10.5 in **Supplementary Fig. 18**.

5- Molecular work: In figure 4 and 5 why different data sets for histone marks are used. Why going from whole embryo histone marks, to neural tube marks and

then to fetal liver. Why not stick with one set of data (whole embryo), especially when comparing gene expression and methylation changes from whole embryo?

We chose to use histone marks derived from neural tube marks and fetal liver because these are – unlike data for the whole embryo - available in ENCODE. Based on reviewer's comments, we changed **Fig. 4** to present all data using fetal liver data sets in the main body of the manuscript and data from neural tubes in **Supplementary Fig. 11** and **12)**. The conclusions derived from both settings are the same with minor differences in the extent of molecular phenotypes. Off note, when we analyzed data sets from heart and facial prominence (also available in ENCODE) we obtained essentially the same conclusions. We choose not to present those data, as they do not provide any additional information.

6- Molecular work: There are 253 up and 819 down regulated genes? Focus is given on the up regulated genes, and the 819 down regulated genes are not elaborated upon? I don't see a good rationale for this. The 819 genes could be critical too.

Because Dnmt3b is a repressor protein, we focused our analysis on up-regulated events as they are more likely to be directly affected by loss of a repressor. In the revised version, we now present results from IPA analysis of 819 genes downregulated genes some of which are also involved in embryogenesis. We added these data as **Supplementary Fig 15.**

7- Molecular work: Wnt9b, Fgf8 and Shh are reported as upregulated in KO embryos and are validated by qPCR and western blot on total embryo samples. Can some IF or in situ be performed to see where in the embryo these genes are upregulated? If lethality is due to these two pathways, are they aberrantly expressed in the organs affected in KO embryos at E11.5?

Please see the response provided to point 2 of reviewer's comments. In addition, we also have phenotypic observation that adult *Dnmt3b*$^{CI/CI}$ show 10% reduction in brain size. We are in a process to better understand the reason for this phenotype. Again, because the manuscript is focused on prenatal development and is already "data-heavy" we choose not to present the data here. Other proposed experiments are interesting but they might be better suited for follow-up studies.

8- Proposed models: The accessory role of dnmt3b should be better defined (ideally show that dnmt3a occupies sites that would normally be occupied by 3b, or show that there is a recruiting function for 3b). What is the accessory role? I understand this may be extensive work for publication of this study at Nat.

Comm. , **but authors at least can more clearly comment on this in the revised manuscript**.

Thank you for this suggestion. We make a comment on this in discussion, please see **page 19**. We would like to point out that we cannot elaborate much more as we need to comply with journal guidelines regarding word limits, number of citations etc.

9- Proposed models: The repressor function should be better defined. How 3b promotes repression non-catalytically? Does it increase the level of H3K27me3 or other repressive marks on the upregulated genes? Does it affect the recruitment of any histone modifying enzymes/complex or affect chromatin accessibility? What is the repressor role? I understand this may be extensive work for publication of this study at Nat. Comm., but authors can more clearly comment on this in the discussion of the revised manuscript, it is very fuzzy to me now.

We do not have any additional data on the mechanism of Dnmt3b-mediated repression. However, we have demonstrated that Dnmt3b is recruited to *Wnt9b* promoter (see response to Reviewer 2). Whether such repression involves recruitment of HDACs or other repressive proteins is unclear. We take a brief note in the discussion, please see **page 21.** However, we are restricted by journal guidelines to extensively elaborate on numerous findings in this manuscript.

Minor points:

1- Global measurement of 5mC levels in WT, CI and KO embryos by dot blot or other methods would be useful to show the effect of Dnmt3b loss on global 5mC level.

As mentioned before, we performed validation experiment of similar type using LC-MS/MS-MRM (**Fig. 1j**).

2- In panels 7i and 7j (where WT or CI 3b are overexpressed in KO cells) the legend in the panel should be properly labeled to show that empty vector control is used.

We have corrected these mistakes.

<u>Reviewer #2:</u>

Major comment:

While the authors nicely demonstrate linkage of wt/CI Dnmt3b expression with Wnt9b/Fgf8, at least one of these interactions should be confirmed. Binding of endogenous or overexpressed/tagged Dnmt3b ChIP should be performed if the authors wish to make the claim that Dnmt3b CI directly regulates Fgf8/Wnt9b, especially due to the previously reported role of Fgf8/Wnt9b in embryonic lethality. This could be even further supported with confirmation of methylation at this locus.

As recommended by the reviewer, we generated and overexpressed FLAG tagged version of Dnmt3b in Dnmt3a$^{-/-}$; *Dnmt3b*$^{-/-}$ lymphoma cell line. Overexpression resulted in gene repression that was accompanied by significant enrichment of Dnmt3b across Wnt9b promoter, most noticeable in region -342 to -127 bp relative to TSS. This result demonstrates that Dnmt3b binds to promoter and represses Wnt9b formally demonstrating this gene is a direct target of Dnmt3b. We present these data in **Fig. 7j.** Because our previous data did not demonstrate any methylation changes in Wnt9b promoter in embryos (**Fig. 7a**), we have not further addressed this point in our overexpression studies in mouse lymphoma cell line.

Minor comments:
Scale in Fig 2D for chrom length (also misspelled)

We corrected these errors.

Fig 2G may be made more convincing by supplementing with genome-wide correlation from all ~800k CpGs to show that CI correlates/clusters better with +/+ at a genome-wide level.

We added this information in **Supplementary Fig. 7.**

Weighted Venns would be helpful in Fig 3a/C to emphasize the fewer number of changes in CI/CI relative to -/-.

We changed data presentation using weighted Venns in **Fig. 3a** and **Fig. 3c** as well as **Supplementary Fig. 9.**

It would be helpful in graphs showing the distribution of changes in genomic features such as enhancers and exons (e.g. Supplemental Fig.7/11) to be adjusted/normalized for their coverage by RRBS. This would make conclusions linked to enrichment at particular regions clearer.

We present the distribution of DMRs identified in *Dnmt3b*$^{-/-}$ and *Dnmt3b*$^{CI/CI}$ embryos within each mentioned element in **Supplementary Fig. 10** and **14**. While we consider

reviewer’s suggestion to be interesting, we feel that an addition of different presentation of elements may make reading of the already complex manuscript more difficult and further dilute basic conclusions.

This manuscript would benefit from general grammar check/proofing.

We appreciate the comment and in response, we extensively edited the manuscript. We believe that the revised version is substantially improved.

Citations:

[1] Chen, T., Tsujimoto, N. & Li, E. The PWWP domain of Dnmt3a and Dnmt3b is required for directing DNA methylation to the major satellite repeats at pericentric heterochromatin. Mol. Cell. Biol. 24, 9048-9058 (2004).

[2] Nishikawa, K. et al. DNA methyltransferase 3a regulates osteoclast differentiation by coupling to an S-adenosylmethionine-producing metabolic pathway. Nat Med 21, 281-287 (2015).

[3] Wu, H. et al. Dnmt3a-dependent nonpromoter DNA methylation facilitates transcription of neurogenic genes. Science 329, 444-448 (2010).

[4] Haney, S.L. et al. Methylation-independent repression of Dnmt3b contributes to oncogenic activity of Dnmt3a in mouse MYC-induced T-cell lymphomagenesis. Oncogene 34, 5436-5446 (2015).

[5] Hlady, R.A. et al. Loss of Dnmt3b function upregulates the tumor modifier Ment and accelerates mouse lymphomagenesis. J Clin Invest 122, 163-77 (2012).

[6] Kiefer, S.M., Robbins, L. & Rauchman, M. Conditional Expression of Wnt9b in Six2-Positive Cells Disrupts Stomach and Kidney Function. PLoS One 7, e43098 (2012).

REVIEWERS' COMMENTS:

Reviewer #1 (Remarks to the Author):

The authors have addressed most of my main comments adequately by performing new experiments or explaining them in the text. Although clear insights into the mechanism is missing (and hopefully will be part of future studies), the revised manuscript seems suitable for publication and will be a nice contribution to the field.

I choose to submit my reviews anonymously. (not signed)

Reviewer #2 (Remarks to the Author):

The authors have done a nice job responding to the prior concerns noted by this reviewer. The inclusion of the DNMT3B ChIP confirmation was an essential piece. I recommend acceptance.

Reviewer #3 (Remarks to the Author):

1. Thus, Dnmt3bCI rescued methylation of ~95% (4,133/4,338) of DMRs detected in Dnmt3b-/- embryos to WT levels.
Technically, one can not "rescue" methylation using an inactive DNMT3b enzyme. A better way to explain this would be "Thus, the presence of Dnmt3bCI resulted in reestablishment of DNA methylation of ~95% (4,133/4,338) of DMRs that was lost in Dnmt3b-/- embryos.

2. Please give a reference to LC-MS/MS-MRM and include the enzymatic digestion step for DNA digestion.

3. Global level of 5-methylcytosine in Dnmt3bCI/CI was reduced relative to both Dnmt3b+/+ and Dnmt3b-/- E11.5 embryos (Fig. 1j). If this is correct what is the mechanism of reestablishment/rescue of DMR methylation?

4. the authors may read and cite previous Dnmt3 null mice work (Ueda et al., 2006 (Development 133, 1183-1192 (2006) doi:10.1242/dev.02293).

RESPONSE TO REVIEWERS

We would like to thank reviewers for their kind remarks regarding the importance of this work toward understanding the role of Dnmt3b catalytic activity in embryogenesis. We are also very thankful for the comments by Reviewer 3 that further helped us to strengthen the revised manuscript.

REVIEWERS' COMMENTS:

Reviewer #1

The authors have addressed most of my main comments adequately by performing new experiments or explaining them in the text. Although clear insights into the mechanism is missing (and hopefully will be part of future studies), the revised manuscript seems suitable for publication and will be a nice contribution to the field.

We appreciate the comments by this Reviewer that contributed to a significant improvement of the manuscript.

Reviewer #2

The authors have done a nice job responding to the prior concerns noted by this reviewer. The inclusion of the DNMT3B ChIP confirmation was an essential piece. I recommend acceptance.

We are very thankful for the comments by this Reviewer that helped us to make our revised manuscript much stronger.

Reviewer #3

1. Thus, Dnmt3bCI rescued methylation of ~95% (4,133/4,338) of DMRs detected in Dnmt3b-/- embryos to WT levels. Technically, one can not "rescue" methylation using an inactive DNMT3b enzyme. A better way to explain this would be "Thus, the presence of Dnmt3bCI resulted in reestablishment of DNA methylation of ~95% (4,133/4,338) of DMRs that was lost in Dnmt3b-/- embryos.

Thank you for this comment. We edited text accordingly.

2. Please give a reference to LC-MS/MS-MRM and include the enzymatic digestion step for DNA digestion.

We included reference as citation number 57 and this information in the manuscript (the Methods section, **page 30**).

3. Global level of 5-methylcytosine in Dnmt3bCI/CI was reduced relative to both Dnmt3b+/+ and Dnmt3b-/- E11.5 embryos (Fig. 1j). If this is correct what is the mechanism of reestablishment/rescue of DMR methylation?

We would like to clarify that global level of 5-methylcytosine in Dnmt3bCI/CI was reduced relative to *Dnmt3b*$^{+/+}$ but not to *Dnmt3b*$^{-/-}$ E11.5 embryos as the small reduction is not statistically significant **(Fig. 1j)**.

We would also like to point out that the global levels of 5-methylcytosine were measured by LC-MS/MS-MRM analysis and reflected averaged out absolute methylation levels of all cytosines in DNA. In contrast, analysis of DMRs presented in **Fig. 2c** was done from data obtained by RRBS on ~800,000 cytosines that were covered in all samples and which reflects methylation readout of ~6% of all cytosines in mouse genome. Consequently, these methods may yield different overall results - reduction in global levels of methylcytosine in *Dnmt3b*$^{CI/CI}$ relative to *Dnmt3b*$^{+/+}$ but rescue in DMRs. The differences in outcome of analysis by two methods can be caused by many factors. The major factor is likely methylation status of 94% of methyl cytosines not covered in RRBS but included in analysis of total methyl cytosine levels. In addition, DMRs calls are defined as ≥30% methylation change in three consecutive cytosines in ≤100 bp but without taking into account the magnitude of change beyond thresholds. E.g., 31% and 100% difference in methylation of individual cytosines are scored the same way because they fulfilled threshold criteria of ≥30% change. Lastly, distribution of methylation changes across the genome is also a contributing factor as it directly affects DMR calls.

4. the authors may read and cite previous Dnmt3 null mice work (Ueda et al., 2006 (Development 133, 1183-1192 (2006) doi:10.1242/dev.02293).

We included this reference as citation number 14.
